# Ontogenetic Changes in the Digestive Capacities of the Naozhou Stock of Large Yellow Croaker (*Larimichthys crocea*)

**DOI:** 10.3390/ani16010120

**Published:** 2025-12-31

**Authors:** Yue Liu, Shu-Pei Huang, Eric Amenyogbe, Ye Yang, Hao-Jie Wang, Zhong-Liang Wang, Jian-Sheng Huang

**Affiliations:** 1 Fishery College, Guangdong Ocean University, Guangdong Marine Fish Science and Technology Innovation Center, Zhanjiang 524025, Chinahuangshupei@stu.gdou.edu.cn (S.-P.H.); yangy659@stu.gdou.edu.cn (Y.Y.); wanghaojie2@stu.gdou.edu.cn (H.-J.W.); leong2006@126.com (Z.-L.W.); 2 Department of Water Resources and Aquaculture Management, University of Environment and Sustainable Development, PMB, Somanya, Ghana; eamenyogbe@uesd.edu.gh; 3 Guangdong Provincial Key Laboratory of Aquatic Animal Disease Control and Healthy Culture, Zhanjiang 524088, China

**Keywords:** Naozhou stock of large yellow croaker *Larimichthys crocea*, natural diet, artificial diet, digestive enzymes, transcriptomics, metabolomics

## Abstract

This study examined the digestive and metabolic responses of Naozhou (NZ) stock large yellow croaker (*Larimichthys crocea*) larvae and juveniles under five developmental feeding stages (DAH3, DAH7, DAH12, DAH19, DAH49) to clarify mechanisms of early nutritional adaptation. Digestive enzyme assays, transcriptome sequencing, and metabolomics were integrated to compare physiological changes across diets. Protease activity increased sharply from DAH7–19 with the introduction of rotifers, Artemia, and copepods, while amylase and lipase activities rose at DAH19–49, reflecting enhanced carbohydrate and lipid utilization during transition to formulated feeds. Transcriptomic analysis showed that differentially expressed genes were enriched in pathways involving protein digestion, lipid and energy metabolism, and cell cycle regulation. The metabolomic analysis further highlighted dynamic changes in amino acid, lipid, carbohydrate, and vitamin metabolism, consistent with transcriptomic findings. The integrated analysis suggests that the coordinated modulation of digestive enzyme activities, gene expression, and metabolite profiles enabled a smooth transition from yolk dependency to live prey feeding and a subsequent use of artificial diets. These findings provide new insights into the early nutritional development of NZ large yellow croaker and provide a scientific basis for the improvement of artificial aquaculture seed production.

## Simple Summary

Young fish are fragile in their early days, and many do not survive because their bodies are still figuring out how to handle different kinds of food. In this study, the young large yellow croaker grows from the stage where they rely on their yolk for nourishment to the point where they can eat tiny live animals and eventually a prepared diet. The study tracked how their digestive system developed and how they used nutrients at each step. The study found out that they start by depending on simple internal food reserves, then quickly strengthen their ability to break down proteins and fats once they begin eating live prey, and finally, they learn to handle more complex diets as their organs mature. The study also finds that changes in digestion were closely tied to shifts in growth, energy use, and organ development. These findings provide new insights into the early nutritional development of NZ large yellow croaker and provide a scientific basis for the improvement of artificial aquaculture seed production.

## 1. Introduction

The large yellow croaker (*Larimichthys crocea*) is an economically important marine species in East Asian countries, particularly China. The human population in China were divided into three geographical groups based on morphology: the Naozhou stock (South China Sea), the Min-yuedeong stock (Eastern South China Sea and Taiwan Strait), and the Diqu stock (East China Sea) [1,2,3]. Although disparate gene pools among these stocks have been described, recent population-based genomic analyses suggest that they were formed through climate changes whereby stock boundaries shifted, and genetic divergence at different latitudes might have also contributed to stock differentiation [4,5,6]. Within this framework, the Naozhou stock (hereafter NZ stock) was shown to be a relatively independent population at both genetic and phenetic levels. Establishing a comprehensive genomic resource is required to advance breeding programs and facilitate conservation [5,7,8,9]. As the NZ stock has a high level of genetic diversity with distinctive phenotypes, it is considered a valuable candidate for selection [8,9]. However, the digestive physiological response to varying diets in this stock is not well understood, presenting a barrier to the development of scientifically formulated diets.

*Larimichthys crocea* undergoes an ontogenetic shift from endogenous to exogenous feeding at 4–6 DAH [10], during which the buccopharyngeal and intestinal tracts and hydrolytic enzymes rapidly develop [11,12,13,14]. Rotifers are introduced at 3–8 DAH [15], followed by Artemia, copepods, and microdiets, reflecting progressive gut maturation [16]. As larvae develop, gastric glands begin secreting acid pepsin and intestinal villi expand [13]. Trypsin and lipase activities rise at 10–20 DAH [17], and juveniles achieve full digestive capacity by ~50 DAH [12,13]. Nevertheless, this stage is sensitive due to immature digestion and fluctuating enzyme activity, contributing to high mortalities [7,18,19]. Balanced larval diets are therefore essential to improve survival and seed quality [16,17].

Live feeds remain standard but present challenges of cost, supply, and pathogen risks [16,17]. Plant protein sources, especially SBM and FSBM, provide sustainable FM replacements, though high SBM levels may induce enteritis and immune activation [20,21]. Fermentation reduces anti-nutrients and improves safety [22,23], while multi-omics studies show plant proteins reshape metabolic and immune pathways [24,25]. Functional additives such as phospholipids and oryzanol enhance survival, enzyme activity, and gut health [25,26,27]. Given phenotypic and genetic distinctions of the NZ stock [5,8,9], diet responses may be stock-specific, yet few studies target this stock [28], underscoring the need to assess its digestive physiology under alternative diets.

Despite advances in genomic characterization of *L. crocea*, the digestive physiology and early nutritional adaptation of the NZ stock remain poorly understood. This knowledge gap presents a critical limitation for stock-specific feed formulation, larval rearing, and selective breeding programs. Early developmental stages of *L. crocea* are particularly sensitive, with digestive enzyme activity, nutrient absorption, and metabolic regulatory mechanisms undergoing rapid ontogenetic transitions. These shifts influence larval survival, feed efficiency, and the success of artificial seed production. While previous studies have examined general digestive development or dietary responses in *L. crocea*, they have rarely accounted for stock identity, and no study has integrated multi-omics approaches to understand the NZ stock’s unique digestive physiology under changing diets. This study represents the first comprehensive multi-omics investigation combining digestive enzyme assays, transcriptomics, and metabolomics to characterize the developmental and diet-dependent digestive physiology of the NZ stock of large yellow croaker. Unlike earlier research that focused on single omics layers or non-stock-specific observations, our integrated approach enables a holistic understanding of how genes, enzymes, and metabolites jointly shape nutritional adaptation from endogenous yolk reliance to live prey feeding and finally to formulated diets. Such a multi-layered perspective is essential for addressing key bottlenecks in NZ stock aquaculture. Stock-specific physiological insights can guide the design of more appropriate formulated diets, improve larval survival and growth, and support selective breeding strategies aligned with the stock’s unique genomic and phenotypic characteristics. This study aims to investigate the stock-specific nutrient requirements needed to maintain intestinal mucosal health in the Naozhou (NZ) stock by integrating enzyme assays with transcriptomic, metabolomic, and differential gene expression analyses. The findings will support the development of sustainable aquaculture feeds that align with the NZ stock’s genomic and phenotypic traits [24,29,30] and guide the formulation of efficient, biosecure diets that promote healthy fish populations and environmentally responsible aquaculture.

## 2. Materials and Methods

### 2.1. Nutritional Enrichment and Artificial Spawning of the NZ Stock

One thousand one hundred and ninety five (1195) wild Naozhou stock broodstock individuals of the NZ stock of large yellow croaker were collected from the coastal waters of Naozhou Island, Zhanjiang, Guangdong Province. After acclimation, the fish were temporarily maintained in land-based concrete tanks (30 m^3^) with controlled rearing water conditions (23–25 °C, 28–30 salinity (ppt), and >6.0 mg·L^−1^ dissolved oxygen) with continuous aeration and daily water exchange at 50% of the tank volume. Shading was provided during acclimation, maintaining a photoperiod of 12 h light and 12 h dark. Only broodstock individuals in good health, with no external injuries and confirmed gonadal development status by biopsy, were selected for the nutritional conditioning phase. Seven hundred and ten (710) parent fish were able to spawn artificially after domestication (including 181 males and 529 females).

To promote gonadal development and enhance reproductive performance, the broodstock was subjected to a 4-week nutritional conditioning period prior to induced spawning. For this, the balanced diet was mainly composed of fresh ragworms (*Nereis* spp.; 60% of the total daily ration) and supplemented with frozen fish (20%) and formulated feed (20%). The fish were fed twice daily (at 08:30 and 17:30) at 3–4% of their body weight, and a vitamin premix (100 mg·kg^−1^ feed) and fish oil (2%) were regularly added to the diet.

Robust and healthy broodstock individuals with gonadal development at stage IV were selected for induced spawning, with a female-to-male ratio of 1:2. Hormonal induction was performed via intramuscular injection at the base of the pectoral fin using a combination of human chorionic gonadotropin (HCG, 800–1000 IU kg^−1^ body weight) and luteinizing hormone-releasing hormone analog (LHRHa, 20–30 µg.kg^−1^ body weight). Females received a single injection, while males were administered half the dosage. After injection, the fish were kept under flow-through and aerated conditions (as above but with a slightly higher temperature of 24–25 °C). Typically, 12–16 h post-injection, the females exhibited abdominal swelling and softness with clear signs of ovulation, while the males reached synchronous spermiation. At this stage, the fish were examined every 2 h to determine the optimal timing for gamete collection. Mature eggs and milt were obtained from females and males, respectively, by gentle abdominal stripping. Fertilization was conducted using the dry method, whereby the eggs and milt were mixed at a ratio of 1:100–150, followed by activation with a small volume of clean seawater. The fertilized eggs were immediately transferred to indoor concrete tanks for incubation. At each developmental stage, three pooled biological replicates (each derived from one rearing tank) were used for both digestive enzyme assays and omics analyses. The number of individuals per replicate varied with fish size smaller larvae required pooled samples of tens of individuals, while larger juveniles required fewer (5–10) fish per replicate.

### 2.2. Egg Hatching and Seedling Cultivation

Hatching buckets were maintained with continuous aeration and a flow-through (as above after injection) and the rearing water was filtered through an 80 μm mesh and exchanged daily at 50% of the tank volume to ensure stable water quality. Hatching was completed within 24 h after fertilization, and by 3 DAH, the larvae had fully absorbed their yolk sacs and initiated exogenous feeding. From 4–7 DAH, larvae were fed 2–3 individuals.mL^−1^ of small rotifers (*Brachionus plicatilis*), which were enriched for 12 h prior to feeding, four times per day. At 8 DAH, 1–2 individuals.mL^−1^ of *Artemia* nauplii were gradually introduced as the main diet, while the proportion of rotifers was reduced. By 12 DAH, *Artemia* completely replaced rotifers. Before the transition from the larval to juvenile stage (around 13 DAH), 2–3 individuals.mL^−1^ of copepod nauplii were gradually introduced. Initially, larvae were co-fed with copepods and *Artemia* to facilitate the diet transition, and by 19 DAH, copepods became the dominant live feed. From 20 DAH onward, the larvae were gradually weaned onto a formulated diet (Supplementary Materials) comprising a combination of live feed and a microdiet. The microdiet contained 45% crude protein and 10% crude lipids (particle size 150–250 μm), of which small amounts were mixed with copepods, progressively increasing until a complete copepod replacement was achieved (Supplementary Materials). Both larvae and juveniles were fed 3–4 times daily at approximately 5% of their body weight. Throughout the rearing period, water quality was maintained at 24–26 °C with the same salinity and dissolved oxygen content as above. Total ammonia nitrogen was kept below 0.2 mg·L^−1^ and nitrite below 0.02 mg·L^−1^. Feces and uneaten feed were removed daily, and probiotics were regularly added to the tanks to stabilize water quality. Growth performance (body length and weight) and survival rate were monitored periodically to evaluate feeding efficiency and success at different dietary stages.

### 2.3. Larval and Juvenile Sampling

Based on the developmental stages and feeding characteristics of the fish larvae and juveniles, five representative sampling points based on number of DAH were established: DAH3 (unfed, yolk sac not fully absorbed), DAH7 (primarily feeding on rotifers, DAH12 (feeding mainly on *Artemia* nauplii), DAH19 (feeding on copepods), and DAH49 (feeding on a formulated diet). Prior to sampling, fish were starved for 24 h to empty the digestive tract and minimize interference from feed residues. At each time point, individuals were anesthetized with MS-222 (100 mg·L^−1^) manufactured by Syndel (a subsidiary of Aquatic Life Sciences, Inc.) Ferndale, MI, United States and was sourced in China., immediately frozen in liquid nitrogen, and stored at –80 °C for subsequent digestive enzyme activity assays and omics analyses.

### 2.4. Digestive Enzyme Activity Assays

The digestive enzyme activities of both larvae and juveniles were measured using commercial assay kits (Nanjing Jiancheng Bioengineering Institute, Nanjing, China). The activities of alkaline phosphatase (AKP), trypsin, pepsin, lipase (LPS), amylase (AMS), and leucine aminopeptidase (LAP) were determined.

For sample preparation, the frozen specimens stored in liquid nitrogen were slowly thawed on ice and accurately weighed. Each sample was homogenized for 3–5 min in ice-cold 0.9% NaCl solution at a ratio of 1:9 (tissue weight (g): buffer volume (mL)) using a glass homogenizer in an ice-water bath until fully disrupted. The homogenates were centrifuged at 2500 r/min for 10 min at 4 °C and the supernatant was collected for enzymatic assays.

The enzyme activities were measured following the manufacturer’s instructions: AKP using p-nitrophenyl phosphate (pNPP) as the substrate, with the absorbance measured at 405 nm; trypsin and pepsin using casein as the substrate, with the absorbance measured at 280 nm; LPS colorimetrically using p-nitrophenyl ester substrates, with the absorbance measured at 420 nm; AMS using soluble starch as the substrate and iodine-starch colorimetry at 660 nm; LAP using L-leucyl-p-nitroanilide as the substrate, with the absorbance measured at 405 nm.

All enzyme activities were expressed as specific activity (U/mg prot), normalized to protein content. Protein concentrations were determined using the Bradford method with Coomassie Brilliant Blue G-250, using bovine serum albumin (BSA) as the standard. Three replicates were sampled at each sampling point, and all assays were conducted at a constant 25 °C to ensure reproducibility and accuracy.

### 2.5. Transcriptome Sequencing and Analysis

#### 2.5.1. RNA Extraction, Library Preparation, and High-Throughput Sequencing

A total of 6 biological replicates were used for the metabolomic analysis. Total RNA was extracted from the fish samples using the TRIzol reagent (Life Technologies, Thermo Fisher Scientific, Waltham, MA, USA) following the manufacturer’s protocol, and genomic DNA was extracted with DNase I (TRANS). RNA integrity and potential DNA contamination were first examined by agarose gel electrophoresis. RNA quality was further assessed using an Agilent 2100 Bioanalyzer (Agilent Technologies, Santa Clara, CA, USA), its purity was determined by a NanoDrop spectrophotometer Guangzhou, China. (OD260/280 and OD260/230 ratios), and the concentration and yield were quantified with a Qubit 2.0 Fluorometer (Toshima City, Japan) [31].

RNA qualifying for analysis was diluted to 1.5 ng.μL^−1^ for cDNA library construction. Poly(A)^+^ mRNA was enriched using oligo(dT)-attached magnetic beads and then fragmented into shorter pieces through ultrasonic disruption. Using the fragmented mRNA as a template, first-strand cDNA was synthesized with reverse transcriptase, followed by second-strand cDNA synthesis with DNA polymerase I and dNTPs after RNase H treatment. The resulting double-stranded cDNA was then purified, subjected to end repair, A-tailing, and adaptor ligation. Target fragments of approximately 200 bp were screened using AMPure XP beads, Santa Clara, CA, USA and sourced in China, followed by PCR amplification and purification to obtain high-quality cDNA libraries.

Three replicate libraries were constructed for each of the five sampling time points. After quality control, all libraries were sequenced on the Illumina NovaSeq 6000 platform, San Diego, CA, USA to generate high-throughput transcriptome data (Reference Genome Information: *Larimichthys crocea* isolate HW-2024, whole-genome shotgun sequencing project. ACCESSION JBIBPI000000000, VERSION JBIBPI000000000.1 and BioProject: PRJNA1169539 with BioSample: SAMN44078122. Web link: https://www.ncbi.nlm.nih.gov/nuccore/JBIBPI000000000.1/, accessed on 23 December 2025). Library preparation and sequencing were conducted by Gene Denovo Biotechnology Co., Ltd. (Guangzhou, China).

#### 2.5.2. Raw Data Quality Control and Filtering

To ensure reliability, image data generated by Illumina NovaSeq 6000 were first processed by the system’s built-in software for base calling and converted into FASTQ format raw sequencing reads. Prior to downstream analyses, strict quality control and filtering of the raw data by removing (1) reads containing adaptor sequences, (2) reads with more than 10% unknown bases (N), (3) reads in which more than 50% of the bases had a Phred quality score (Q) below 20, and (4) low-complexity reads composed of a single nucleotide (e.g., continuous poly-A). The resulting high-quality sequences were defined as clean reads and used as the basis for subsequent analyses. In addition, the quality of the clean reads was evaluated using FastQC software Version 0.12.0 and in-house scripts, assessing indicators such as Q20, Q30, and GC content to ensure the accuracy and reliability of the sequencing data.

#### 2.5.3. Differentially Expressed Gene (DEG) Analysis

After transcriptome sequencing, reads were mapped to the reference genome, gene expression levels were quantified, and DEGs were identified using DESeq2 (version 1.30.0) [32]. Raw read count data were first normalized to eliminate any biases caused by sequencing depth and gene length. A negative binomial statistical model was applied to assess significant expression differences between groups. Correction for multiple comparisons was performed using the Benjamini–Hochberg method to obtain the false discovery rate (FDR), thereby reducing false positives. Genes with FDR < 0.05 and |log_2_(FC)| > 1 (i.e., fold change, FC > 2 and statistically significant after FDR correction) were defined as DEGs. Subsequent multidimensional functional analyses were then performed including volcano plots to visualize the magnitude and significance of gene expression changes, a Gene Ontology (GO) enrichment analysis to identify significantly enriched categories in biological processes (BP), molecular functions (MF), and cellular components (CC), and a Kyoto Encyclopedia of Genes and Genomes (KEGG) pathway analysis to determine significantly enriched pathways related to metabolism, signal transduction, and physiological functions.

#### 2.5.4. Quantitative Real-Time PCR (RT-qPCR)

To validate the reliability of the RNA sequencing (RNA-seq) data, ten DEGs were randomly selected for the RT-qPCR analysis, with β-actin acting as the internal reference gene. Gene-specific primers were designed based on the cDNA sequences using Primer Premier version 5.0 (Table 1) and synthesized by Sangon Biotech Co., Ltd. (Shanghai, China). RT-qPCR assays were performed on an ABI QuantStudio 3 Real-Time PCR system, Waltham, MA, USA and sourced in China. Each 10 μL of reaction mixture contained 5 μL of 2× PerfectStart Green qPCR SuperMix, 3.2 μL of nuclease-free water, 1 μL of cDNA template, and 0.4 μL each of both forward and reverse primers (10 µmol·L^−1^). Amplification was carried out through initial denaturation at 95 °C for 30 s, 40 cycles at 95 °C for 5 s and at 60 °C for 15 s, then at 95 °C for 15 s, 60 °C for 1 min, and 95 °C for 1 s. All reactions were performed in triplicate, and the Ct value was analyzed using the 2^−ΔΔCt^ method [33].

### 2.6. Metabolomic Sequencing and Analysis

#### 2.6.1. Metabolite Extraction

Samples were thawed slowly at 4 °C, and an appropriate amount of tissue (0.3 g) was transferred into pre-cooled methanol/acetonitrile/water solution (2:2:1, *v*/*v*). After vortexing, samples were subjected to low-temperature ultrasonication for 30 min, followed by incubation at −20 °C for 10 min. The mixture was then centrifuged at 14,000× *g* for 20 min at 4 °C, the supernatant was collected and vacuum-dried, and these dried extracts were reconstituted in 100 μL of acetonitrile/water solution (1:1, *v*/*v*), vortexed, and centrifuged at 14,000× *g* for 15 min at 4 °C. The resulting supernatant was used for the liquid chromatography–mass spectrometry (LC-MS) analysis.

Chromatographic separation was performed using an Agilent 1290 Infinity LC UHPLC system equipped with a HILIC column. A 2 μL aliquot of each sample was injected with an autosampler, and separation was carried out under gradient elution with a flow rate of 0.5 mL.min^−1^ and a column temperature of 25 °C. The mobile phases consisted of (A) water +25 mM ammonium acetate +25 mM ammonium hydroxide, and (B) acetonitrile and the gradient program followed 95% B for minute 0–0.5, a linear decrease in B from 95% to 65% for minute 0.5–7, a linear decrease in B from 65% to 40% for minute 7–8, 40% B for minute 8–9, a linear increase in B from 40% to 95% for minute 9–9.1, and 95% B for minute 9.1–12. During this time, the samples were kept in the autosampler at 4 °C. To minimize signal drift, samples were randomly analyzed, with quality control (QC) samples inserted into the sequence to monitor instrument stability and data reliability.

#### 2.6.2. Identification of Differential Metabolites

The method was slightly modified based on the method described by [34]. Raw LC-MS data were converted into MzML format using ProteoWizard software (v3.0.8789), and peak detection, retention time alignment, and peak area extraction were performed using XCMS software (Version 3.7.0) [35], with parameter settings as follows: for peak picking centWave *m*/*z* tolerance = 10 ppm, peakwidth = c(10, 60), prefilter = c(10, 100); for peak grouping bw = 5, mzwid = 0.025, minfrac = 0.5. The extracted data were checked for completeness, and total ion current (TIC) normalization was applied to ensure comparability across samples and metabolites. Metabolites with more than 50% missing values were excluded from further analysis and remaining missing values were imputed using the K-Nearest Neighbors (KNN) method [36,37]. Outliers were removed, and normalized peak areas were used for subsequent statistical analyses.

A data matrix was generated containing retention time (RT), mass-to-charge ratio (*m*/*z*), and peak intensity information. Metabolite annotation was achieved through matching them against public databases including MassBank, Metlin, and MoNA, in combination with an in-house secondary mass spectrometry database.

To evaluate biological reproducibility among samples, a correlation analysis was performed, followed by a principal component analysis (PCA) and orthogonal partial least squares-discriminant analysis (OPLS-DA) comparing experimental and QC samples [38]. A 200-time permutation test was used to assess potential model overfitting. Differential metabolites between groups were identified based on the intersection of three criteria: FC > 1, *p* < 0.05 (Student’s *t*-test), and variable importance in projection (VIP) > 1 derived from the OPLS-DA. Pathway annotation of differential metabolites was conducted using the KEGG database and identified metabolites were classified using the KEGG Compound and Human Metabolome Database (HMDB) for compound category statistics [39].

### 2.7. Data Analysis

The enzyme activity data were statistically analyzed using GraphPad Prism 10 (GraphPad Software, Boston, MA, USA, www.graphpad.com). Differences among groups were evaluated by one-way analysis of variance (ANOVA), followed by post hoc multiple comparisons when the assumption of homogeneity of variance was met. A significance level of *p* < 0.05 was used and all results are presented as mean ± standard deviation (SD).

## 3. Results

### 3.1. Diet-Based Effects on Digestive Enzyme Activities

Changes in the digestive enzyme activities of NZ large yellow croaker at different developmental stages are shown in Table 2. As indicated, AKP, LAP, AMS, pepsin, and trypsin in the DAH49 group (formulated diet stage) were significantly higher than those in the DAH3, DAH7, DAH12, and DAH19 groups (*p* < 0.05). During the live feed stages (DAH7–DAH19), trypsin activity was significantly higher than in the DAH3 group (unfed stage) (*p* < 0.05), suggesting that ingestion of exogenous feed markedly enhanced protein digestion capacity. In contrast, LPS activity did not differ significantly among developmental stages (*p* > 0.05).

### 3.2. Transcriptome Analysis

#### 3.2.1. RNA-Seq Quality Assessment

Transcriptome sequencing was performed for all groups, with three biological replicates per group. A total of 676,738,462 bp of raw sequencing data were generated and after QC and removal of low-quality reads, 676,519,612 bp of clean data were retained (Table 3). A base quality analysis showed that Q20 values exceeded 99.36% and Q30 values exceeded 97.57% for all samples, indicating high sequencing accuracy. The GC content ranged from 47.63% to 48.64%, falling within a reasonable range. Overall, the data quality was sufficient for subsequent transcriptome assembly and differential expression analysis.

#### 3.2.2. Identification of DEGs

**Figure 1:**
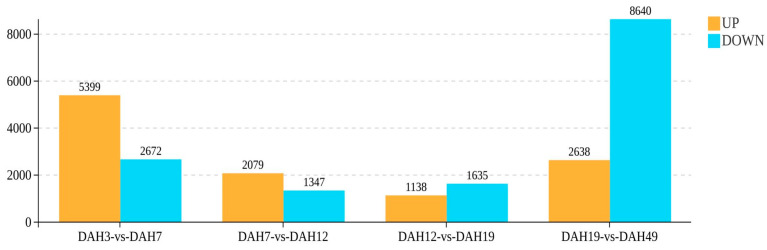
Bar chart showing identified DEGs.

Significant DEGs were detected in all pairwise comparisons across the different feeding stages (Figure 1) comprising 8071 between DAH3 and DAH7 (5399 upregulated and 2672 downregulated), 3426 between DAH7 and DAH12 (2079 upregulated and 1347 downregulated), 2773 between DAH12 and DAH19 (1138 upregulated and 1635 downregulated), and the most pronounced difference between DAH19 and DAH49 with 11,278 DEGs (2638 upregulated and 8640 downregulated).

Overall, throughout development and across varying diets, the number of significant DEGs decreased, followed by an increase, and then decreased again. During early developmental stages (DAH3–DAH12), the number of upregulated genes was consistently higher than downregulated genes, suggesting activation of genes related to digestive system development and adaptation to exogenous nutrition. In contrast, during later developmental stages (DAH12–DAH49), the number of downregulated genes was higher, particularly during the transition from DAH19 to DAH49, indicating that certain genes with high expression in early developmental stages were progressively suppressed.

#### 3.2.3. Gene Ontology Analysis of Differentially Expressed Genes

**Figure 2:**
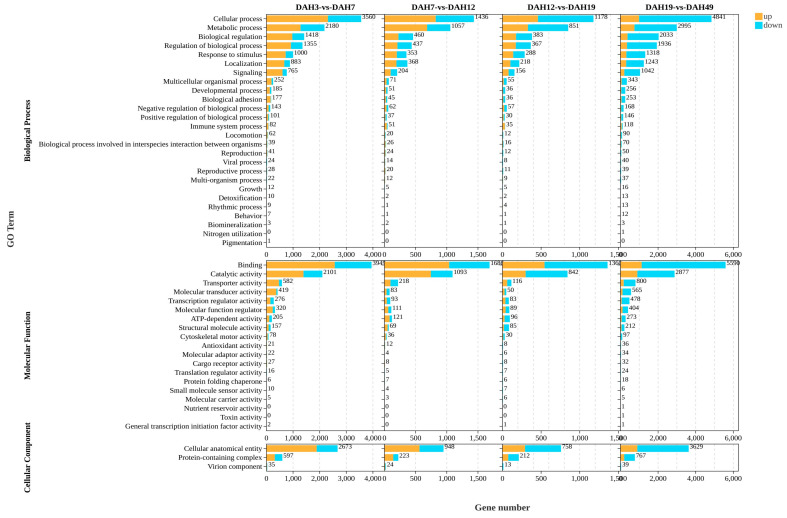
The GO enrichment analysis of the DEGs.

To investigate the potential biological functions of DEGs and their roles in larval–juvenile development of the NZ stock, a GO enrichment analysis was performed (Figure 2). The results showed that DEGs from all five feeding stage comparisons were classified into the three major GO categories (BP, MF, and CC), encompassing a total of 48 subcategories.

For BP, DEGs were mainly enriched in fundamental biological activities such as cellular and metabolic processes, biological regulation, and responses to stimuli. For MF, enrichment was dominated by binding and catalytic activity, indicating that many DEGs are involved in substrate binding and catalytic reactions. For CC, DEGs were primarily associated with cellular anatomical structures and protein-containing complexes. Overall, MF DEGs contained the most enriched genes, suggesting that enzyme activity and related molecular binding functions play pivotal roles for the larval-juvenile stages in regulating digestive metabolism and development across different feeding stages.

#### 3.2.4. Kyoto Encyclopedia of Genes and Genomes Pathway Enrichment Analysis

**Figure 3:**
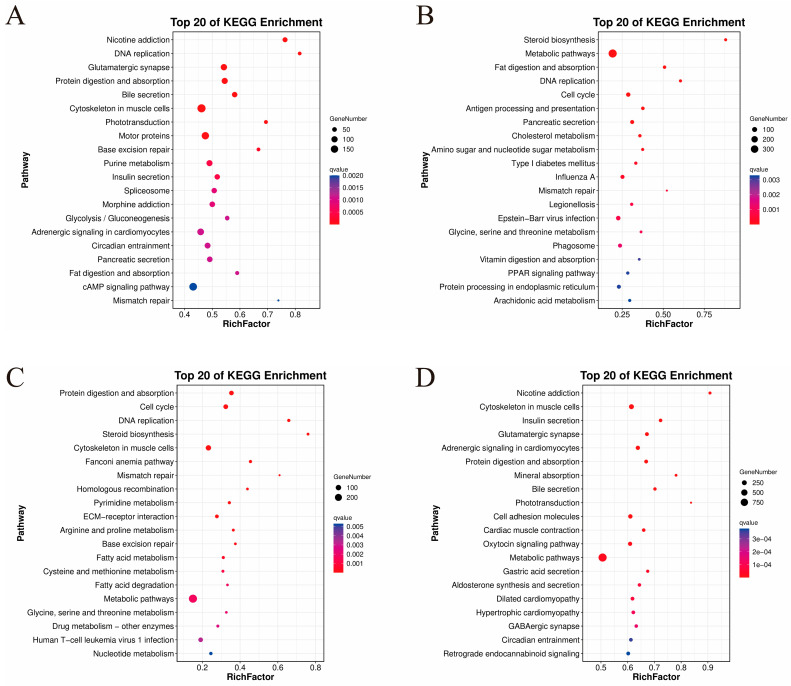
The KEGG enrichment analysis comparing the feeding stages. (**A**) DAH3 and DAH7, (**B**) DAH7 and DAH12, (**C**) DAH12 and DAH19, (**D**) DAH19 and DAH49.

To investigate the functional roles of DEGs during the larval–juvenile development stages of the NZ stock, DEGs were mapped to the KEGG database for an enrichment analysis (Figure 3).

When comparing DAH3 and DAH7 (Figure 3A), 366 enriched pathways were identified, of which 47 were significantly enriched. These were mainly associated with muscle cytoskeleton, motor proteins, glutamatergic synapse, and protein digestion and absorption, which are closely related to cell proliferation, tissue differentiation, and energy supply, providing the material and energy required for early larval development.

When comparing DAH7 and DAH12 (Figure 3B), 364 pathways were enriched, 37 of which were significantly enriched. These were primarily related to metabolic pathways, the cell cycle, and pancreatic secretion, and the associated genes were involved in the metabolism of steroids, carbohydrates, and lipids, and lipid digestion and bile secretion (digestive processes), as well as immune-related pathways including antigen processing and presentation. These changes ensured efficient nutrient use and supported the establishment of the immune defense system.

Comparing DAH12 and DAH19 (Figure 3C), 360 pathways were enriched, with 47 showing significant enrichment. These were mainly associated with muscle cytoskeleton, extracellular matrix–receptor interactions, and pyrimidine metabolism, suggesting that larvae-enhanced protein digestion and absorption support rapid increases in body length and weight, cell cycle regulation, and DNA replication ensure normal cell proliferation and division, and genome stability is maintained by genetic material repair. These processes are all critical for somatic growth and cellular development.

Comparing DAH19 and DAH49 (Figure 3D), 371 enriched pathways were identified, of which 99 were significantly enriched. These included metabolic pathways, cell adhesion molecules, muscle cytoskeletons, oxytocin signaling pathways, and adrenergic signaling in cardiomyocytes, driving the development of major organs (nervous and circulatory systems) and nutrient digestion and utilization.

Overall, the identified DEGs in both larvae and juveniles were mainly enriched in pathways related to digestion and absorption, energy metabolism, and tissue development. Early developmental stages were characterized by the establishment of nutrient metabolism and absorption, whereas later developmental stages also included cell cycle regulation, signal transduction, and the functional maturation of multiple organ systems. This indicates that both larvae and juveniles adapt to sudden environmental changes through coordinated regulation of diverse metabolic and signaling pathways throughout development.

#### 3.2.5. Quantitative Reverse Transcription Polymerase Chain Reaction Validation Results

**Figure 4:**
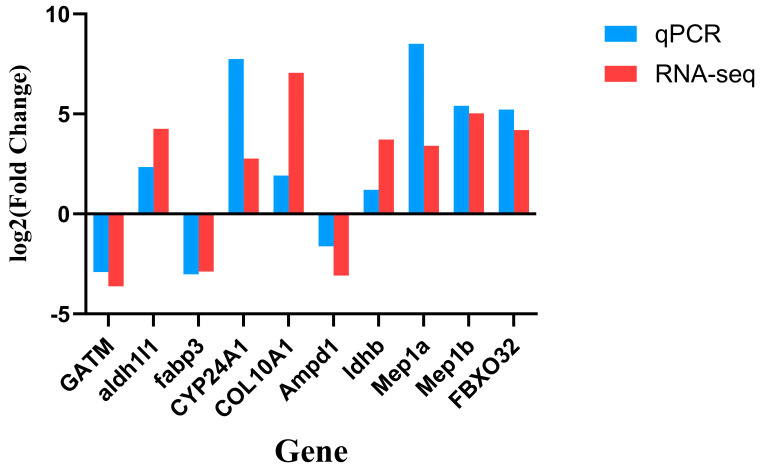
Verification of DEGs by qRT-PCR.

To validate the accuracy of the RNA-seq data, 10 genes were randomly selected from each developmental stage for the RT-qPCR analysis. The results showed that the expression trends of these genes were highly consistent with those obtained from RNA-seq (Figure 4), supporting the reliability and reproducibility of the transcriptome sequencing data used in this study.

### 3.3. Metabolomic Results

#### 3.3.1. Sample Quality and Multivariate Statistical Analysis

**Figure 5:**
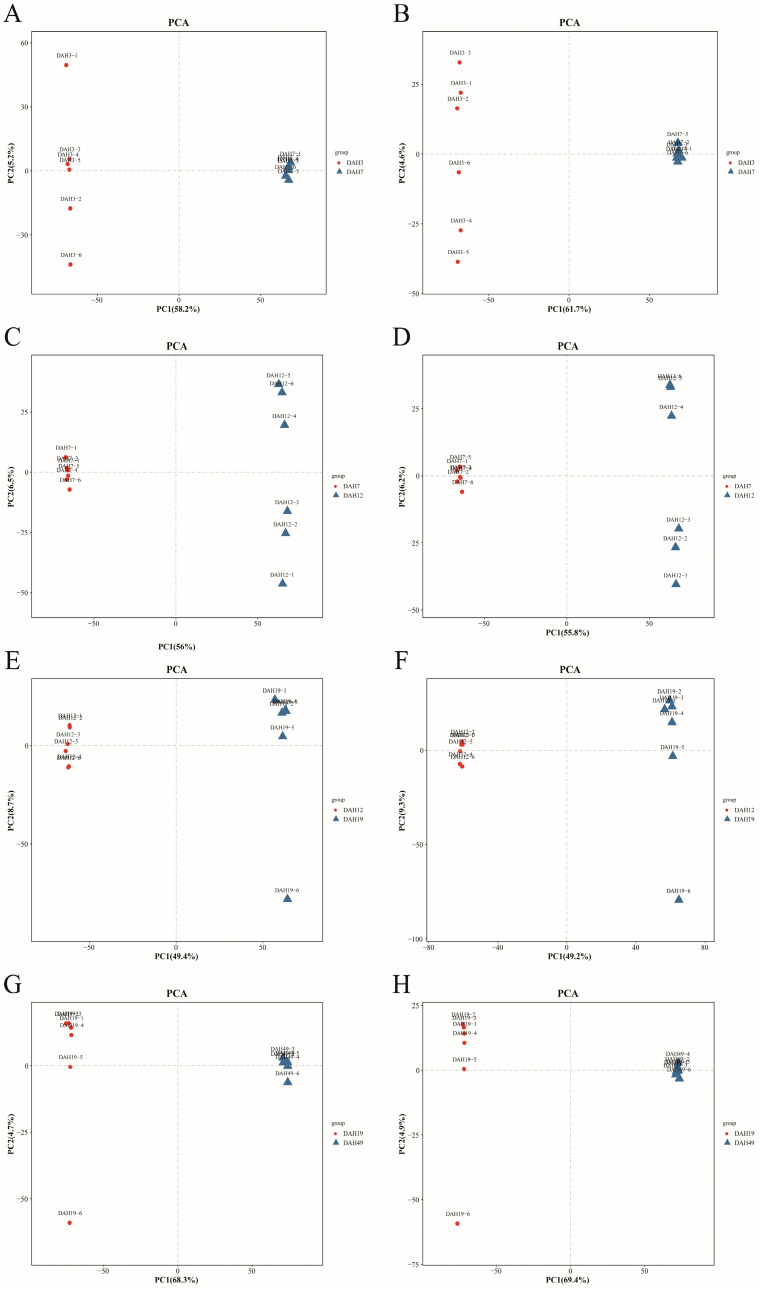
PCA scores. (**A**) 3DAH-vs-7DAH Positive Ion Mode, (**B**) 3DAH-vs-7DAH Negative Ion Mode; (**C**) 7DAH -vs-12DAH Positive Ion Mode, (**D**) 7DAH -vs-12DAH Negative Ion Mode; (**E**) 12DAH -vs-19DAH Positive Ion Mode, (**F**) 12DAH-vs-19DAH Negative Ion Mode; (**G**) 19DAH -vs-49DAH Positive Ion Mode, (**H**) 19DAH-vs-49DAH Negative Ion Mode.

A PCA was performed for all sample groups under both positive and negative ion (NEG) modes. The results showed that the QC samples clustered closely together (Figure 5), indicating data stability and reproducibility, validating the reliability of subsequent metabolomic analyses.

#### 3.3.2. Orthogonal Partial Least Squares Discriminant Analysis

**Figure 6:**
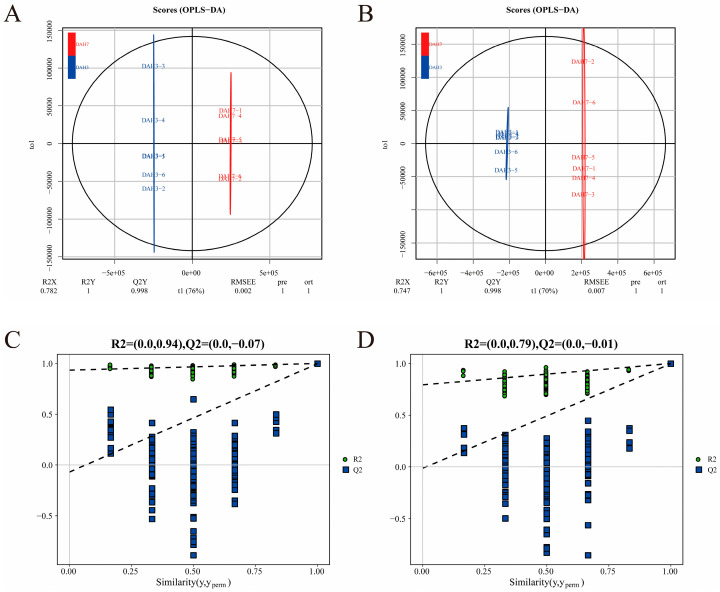
OPLS-DA scores and ranking for DAH3 and DAH7. Panels (**A**,**C**) represent the positive ion mode; panels (**B**,**D**) represent the negative ion mode.

**Figure 7:**
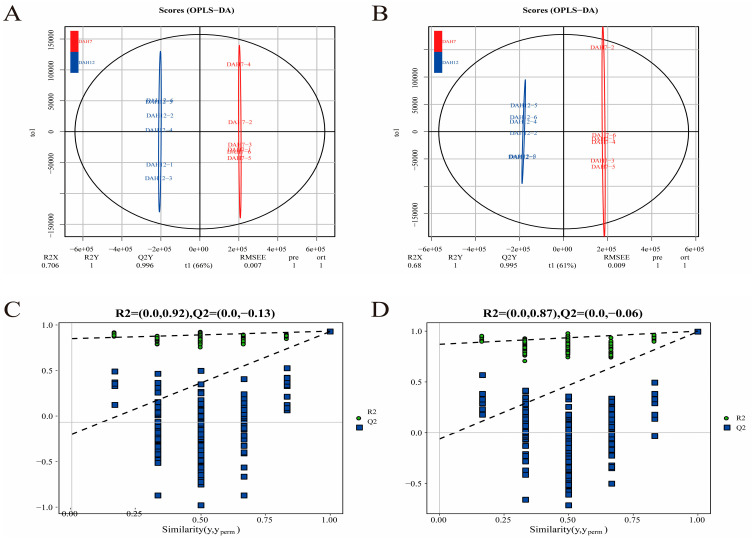
OPLS-DA scores and ranking for DAH7 and DAH12. Panels (**A**,**C**) represent the positive ion mode; panels (**B**,**D**) represent the negative ion mode.

**Figure 8:**
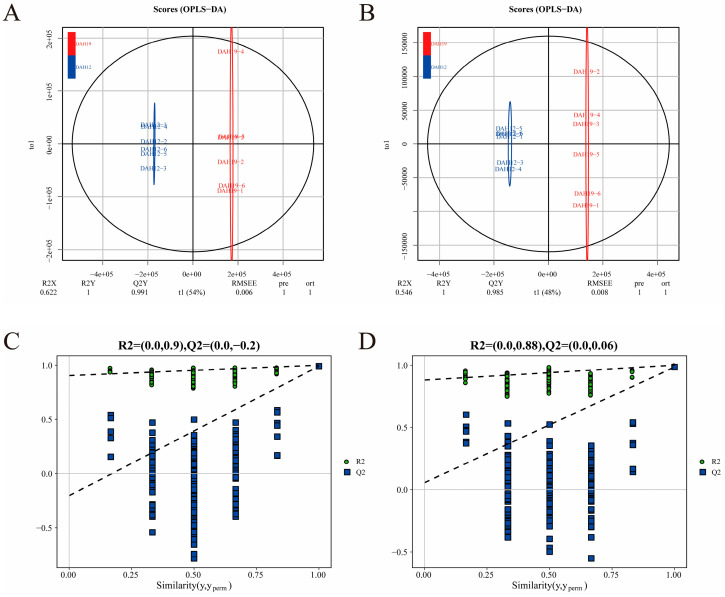
OPLS-DA scores and ranking for DAH12 and DAH19. Panels (**A**,**C**) represent the positive ion mode; panels (**B**,**D**) represent the negative ion mode.

**Figure 9:**
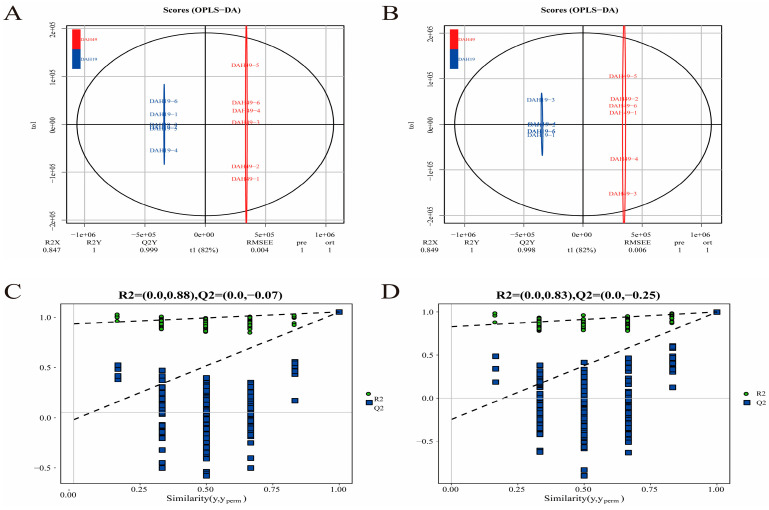
OPLS-DA scores and ranking for DAH19 and DAH49. Panels (**A**,**C**) represent the positive ion mode; panels (**B**,**D**) represent the negative ion mode.

To investigate the metabolic profile differences among feeding groups, OPLS-DA models were constructed. Under both positive and negative ion modes, all groups exhibited clear distinction (Figure 6, Figure 7, Figure 8 and Figure 9A,B), indicating significant metabolic differences associated with larval-juvenile development and dietary changes.

To verify the robustness and reliability of the models, 200 permutation tests were conducted by randomly shuffling the class variable Y. The R^2^ and Q^2^ of the randomized models were markedly lower than those of the original models (Figure 6, Figure 7, Figure 8 and Figure 9C,D), and the Q^2^ regression line and *y*-axis intercept were less than zero. This demonstrates the reliability of the OPLS-DA models through good fitting performance and predictive ability, validating its use for the identification and functional interpretation of differential metabolites.

#### 3.3.3. Screening and Identification of Differential Metabolites

**Figure 10:**
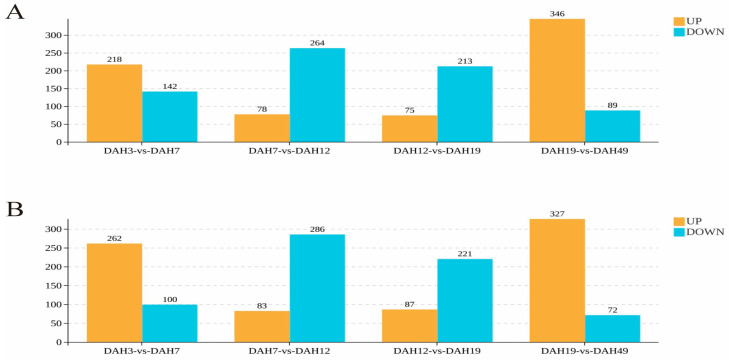
Statistical chart of the quantity of differential metabolites. (**A**) positive ion mode, (**B**) negative ion mode.

In the positive ion mode (POS; Figure 10A), 360 differential metabolites were identified between DAH3 and DAH7 (218 upregulated and 142 downregulated), 342 between DAH7 and DAH12 (78 upregulated and 264 downregulated), and 288 between DAH12 and DAH19 (75 upregulated and 213 downregulated). The most pronounced differences were observed between DAH19 and DAH49 with 435 identified differential metabolites, of which 346 were upregulated and 89 were downregulated.

In the NEG mode (Figure 10B), 362 differential metabolites were identified between DAH3 and DAH7 (262 upregulated and 100 downregulated), 369 between DAH7 and DAH12 (83 upregulated and 286 downregulated), 308 between DAH12 and DAH19 (87 upregulated and 221 downregulated), and 399 between DAH19 and DAH49 (327 upregulated and 72 downregulated).

Overall, the number of differential metabolites, as well as those up- and downregulated, represented stage-specific changes. From DAH3 to DAH7, the number of upregulated metabolites was greater than downregulated ones, from DAH7 to DAH19, downregulated metabolites predominated, and from DAH19 to DAH49, the number of upregulated metabolites increased again. This pattern reflects the dynamic metabolic shifts in both the larvae and juveniles during different developmental stages and feeding conditions, transitioning from early active regulation to mid-stage adaptation to enhanced utilization in later stages.

#### 3.3.4. Differential Metabolite and Pathway Analysis

**Figure 11:**
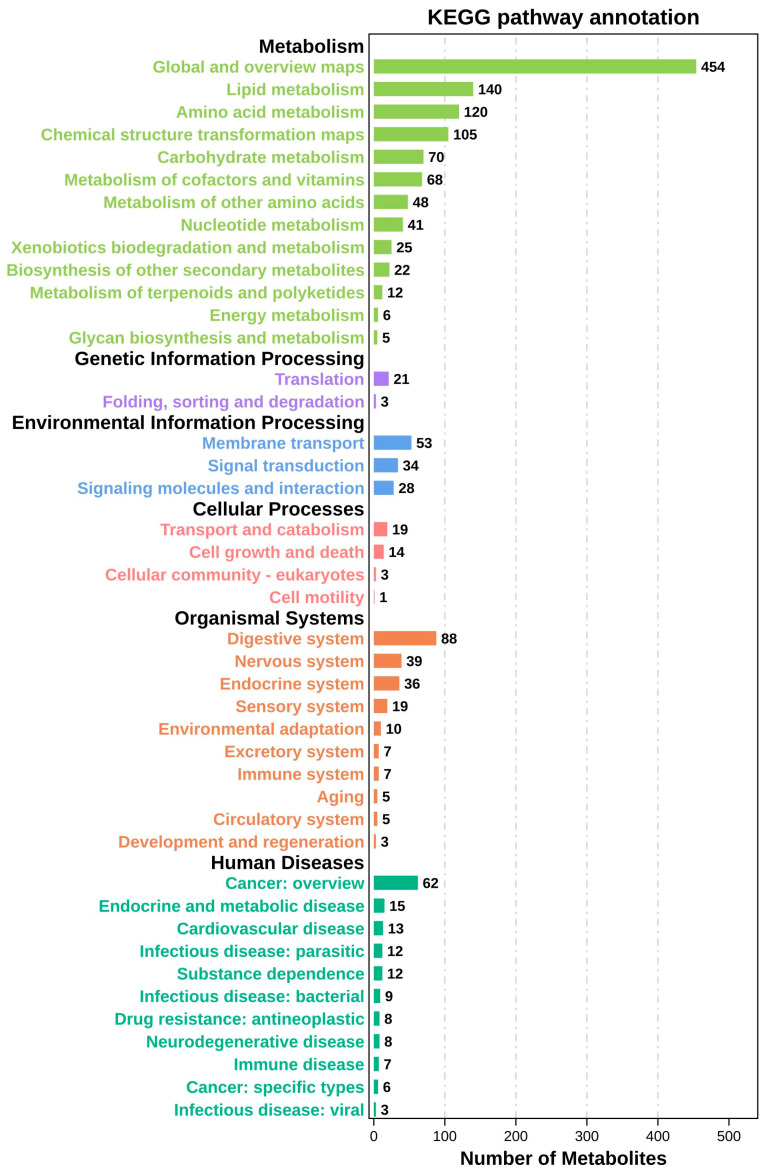
KEGG pathways of differential metabolites.

To investigate the metabolic characteristics of larvae at different feeding stages, a KEGG pathway enrichment analysis was performed on the identified differential metabolites (Figure 11). These differential metabolites were mainly grouped into six major biological categories, including metabolism, genetic information processing, environmental information processing, cellular processes, organismal systems, and human diseases. These involved multiple key pathways, including the metabolism of amino acids, lipids, carbohydrates, nucleotides, cofactors, and vitamins, as well as membrane transport and the digestive system.

From DAH3 to DAH7, differential metabolites were significantly enriched in amino acid and lipid metabolism pathways. In particular, elevated levels were observed for taurochenodeoxycholate, taurolithocholate, isoleucine, taurine, N-acetyl-L-phenylalanine, oleic acid, 1-palmitoyl-sn-glycero-3-phosphocholine, and creatine, while lower levels were observed for palmitic acid and 1-stearoyl-2-hydroxy-sn-glycero-3-phosphocholine (Table 4). This suggests that larvae in the early developmental stages relied heavily on amino acid and lipid metabolism to support rapid development. From DAH7 to DAH12, differential metabolites were significantly enriched in nucleotide and lipid metabolism pathways. In particular, hypoxanthine levels increased, while choline cation, 1-palmitoyl-sn-glycero-3-phosphocholine, adenine, leucine, 1-hexadecyl-sn-glycero-3-phosphocholine, trimethylamine N-oxide, cadaverine, taurochenodeoxycholate, 8S-hydroxy-5Z,9E,11Z,14Z-eicosatetraenoic acid, taurocyamine, and pantothenate significantly decreased (Table 5), reflecting gradual improvement of nucleotide synthesis and digestive functions accompanied by decreased activity in certain lipid and amino acid metabolic pathways. From DAH12 to DAH19, differential metabolites were primarily associated with energy and vitamin metabolism where acetyl-DL-carnitine, vitamin C, and taurochenodeoxycholate levels increased, while leucine, nicotinamide, lithocholic acid, pantetheine, succinate, and glycocholic acid levels decreased (Table 6). This indicates that vitamin and energy metabolism pathways were activated in the larvae to aid in rapid growth, even though some amino and bile acid metabolic activities were reduced. From DAH19 to DAH49, differential metabolites were enriched in lipid and steroid metabolism pathways where nicotinamide, glycochenodeoxycholate, trimethylamine N-oxide, glycocholic acid, thiamine cation, 11-dehydrocorticosterone, taurolithocholate, taurocholate, isoleucine, parsilin, and taurine levels significantly increased, while deoxycholic acid levels decreased (Table 7). This suggests that after transitioning to a formulated diet, lipid metabolism and bile acid biosynthesis were enhanced by the larvae, thereby improving digestion and supporting organ system development.

Overall, the KEGG enrichment analysis revealed that as feeding regimes shifted during larval development, metabolic regulation transitioned from amino acid and energy metabolism in early stages toward lipid and vitamin metabolism, and multi-system coordinated development in later stages. This reflects the dynamic metabolic adaptations underlying rapid growth and nutritional adjustment.

**Figure 12:**
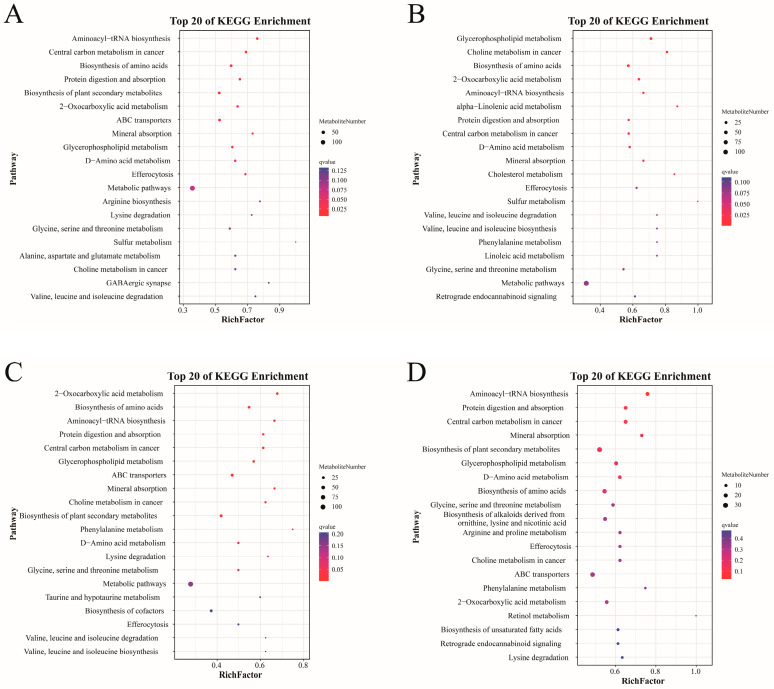
The top 20 KEGG enrichment bubble charts. (**A**) DAH3 and DAH7, (**B**) DAH7 and DAH12, (**C**) DAH12 and DAH19, (**D**) DAH19 and DAH49.

At different larval-juvenile developmental stages, the differential metabolites exhibited distinct pathway enrichment patterns (Figure 12). From DAH3 to DAH7, 152 pathways were enriched (27 significantly), mainly involving aminoacyl-tRNA, amino acid, and plant secondary metabolite biosynthesis, carbon and 2-oxocarboxylic acid metabolism, and protein digestion and absorption. From DAH7 to DAH12, 137 pathways were enriched (28 significantly), primarily glycerophospholipid, 2-oxocarboxylic acid, and α-linolenic acid metabolism, and amino acid biosynthesis. From DAH12 to DAH19, 138 pathways were enriched (25 significantly), mainly 2-oxocarboxylic acid metabolism, amino acid and aminoacyl-biosynthesis, and protein digestion and absorption. From DAH19 to DAH49, 151 pathways were enriched (17 significantly), mainly related to aminoacyl-tRNA and plant secondary metabolite biosynthesis, protein digestion and absorption, carbon metabolism, and mineral absorption.

Overall, metabolic pathway enrichment exhibited stage-specific shifts with the early stages dominated by amino acid and carbon metabolism, the midway stages showing progressively enhanced lipid metabolism, and the later stages transitioning toward improved nutrient absorption and multi-system metabolism.

**Figure 13:**
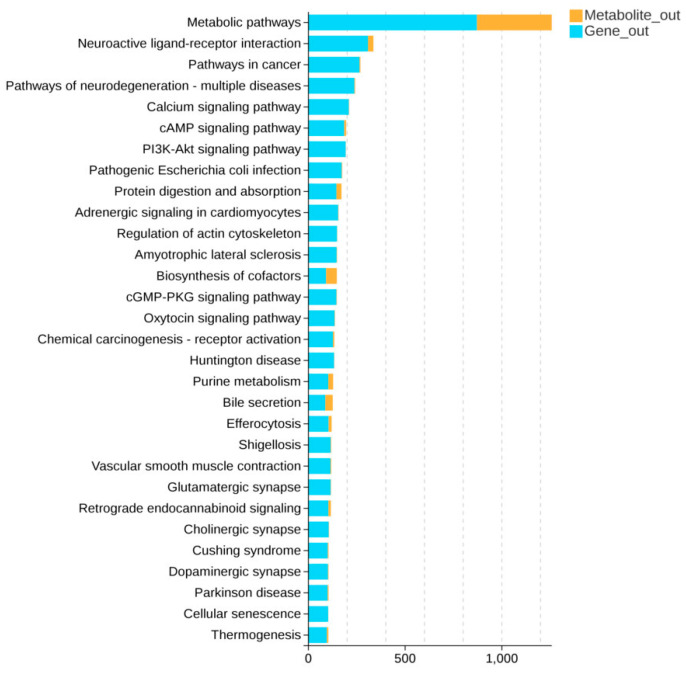
Pathway Association Analysis Bar Chart. Note: The vertical axis represents different metabolic pathway names, while the horizontal axis indicates the number of metabolites or genes enriched in that pathway. Gene_out (number): The number of genes enriched in this pathway within the gene set used for analysis. (The number in parentheses in the header indicates the number of genes with KEGG annotation in the gene set used for analysis). Metabolite_out (number): Number of metabolites enriched in this pathway within the analyzed metabolite set. (The number in parentheses in the header indicates: Number of KEGG-annotated metabolites in the analyzed metabolite set).

The joint analysis revealed significant alterations in metabolic pathways during the early developmental stages of the Dazhu strain of yellow croaker, with particularly pronounced changes in the “metabolic pathway” category. As shown in Figure 13, nearly 870 genes were enriched in this pathway, significantly higher than the enrichment level of metabolites, suggesting the potential existence of a complex metabolic regulatory network. In contrast, pathways such as “neurological ligand-receptor interactions,” “cAMP signaling,” and “PI3K-Akt signaling” exhibited moderate enrichment of metabolites and genes but demonstrated relatively limited regulatory effects. Furthermore, pathways like “Dopaminergic Synapse” and “Cholinergic Synapse” showed lower involvement. These findings suggest that “metabolic pathways” play a key regulatory role in transcriptomics-metabolomics association analysis and represent the most prominent pathways for metabolic changes during early developmental stages.

**Figure 14:**
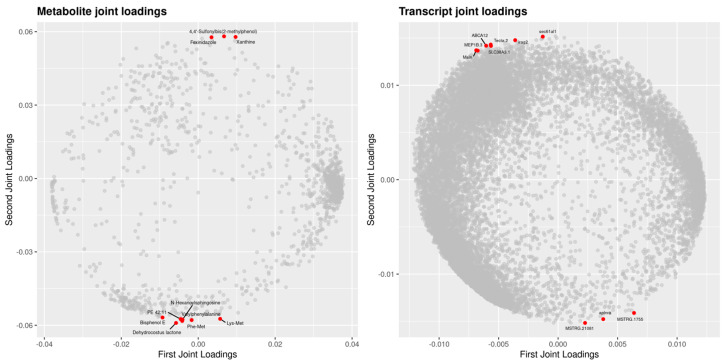
O2PLS Loading Plots for Two Groups of Data. Note: Both plots display loadings for elements in the joint component. The left plot represents metabolomics data, while the right plot represents transcriptomics data. Each point corresponds to a gene or a metabolite. The *x*-axis represents the first dimension of the joint component, and the *y*-axis represents the second dimension. A higher absolute value of an element in the coordinate system indicates a stronger association between that element and the other omics data.

The analysis of the results further indicate (Figure 14): Genes farther from the origin (such as ABCA12, MEP1B.3, Msln, SLC38A3.1, sec61al1) and metabolites (e.g., 4,4′-Sulfonylbis(2-methylphenol), Fexinidazole, Xanthine) indicate stronger associative effects on another omics dataset (metabolome or transcriptome), potentially serving as key factors in cross-omics regulation. The dispersed distribution of genes and metabolites suggests significant variations in contribution across different variables within the joint analysis. For instance, certain genes exhibit high loadings on both principal components, while some metabolites show lower loading values, potentially reflecting their lesser contribution to the analysis or interference from other factors.

## 4. Discussion

### 4.1. Diet-Based Effects on Digestive Enzyme Activities

In this study, the digestive enzymatic activity, transcriptomic expression, and metabolomics of larvae and juveniles of the NZ stock of *L. crocea* were closely associated with diet. Protease activity was significantly enhanced in the early-stage post-exogenous feeds (DAH7–19) for live high protein prey digestion, particularly during the copepod diet and formulation stage, indicating better carbohydrate and lipid utilization. These enzyme-driven changes align with the transcriptomic enrichment of protein digestion, lipid metabolism, and energy pathways, as well as changes in amino acid, lipid, and carbohydrate content. The integration of the enzymatic, transcriptomic, and metabolomic data highlights the adaptive flexibility of *L. crocea* regarding diet shifts during early developmental stages.

Previous studies have suggested that ingesting live prey partly influences the digestive enzyme activity of fish larvae [40,41]. Accumulating evidence indicates that exogenous contributions are minimal to the digestive enzyme system of larvae and juveniles, which is predominantly regulated by endogenous genetic mechanisms and the progression of digestive organ development [42,43]. For the red drum (*Sciaenops ocellatus*), the presence of digestive enzymes precedes the onset of initial feeding behavior, indicating that gene expression regulation is the core mechanism governing the early physiological preparation for digestion [44]. Enzyme secretion depends on developmental stage, nutritional requirements, and life mode [45,46], with key changes occurring at diet (yolk to feed) and developmental (larvae to juvenile) shifts [47,48]. Together, these studies indicate that enzyme activity is influenced by both genetic and environmental effects. At DAH3-7, AKP, LAP, LPS, AMS, trypsin, and pepsin levels remained low, supporting yolk nutrient contribution, as seen in long-spine porcupinefish (*Diodon holocanthus*) where these maternal reserves were used [49]. These basal levels primarily mobilize the yolk but provide pre-adaptation for subsequent feeding stages. During DAH7-19, the enzyme levels increased, indicating the transition into exogenous feeding. Here, trypsin and pepsin increased with protein ingestion, LAP improved the absorption of amino acids, and AMS and LPS were adjusted for the digestion of carbohydrates and lipids. In other marine species, increased trypsin appears to be an adaptation for incomplete gastric function [50,51,52]. Furthermore, essential amino acids have been shown to support both protein synthesis and energy production [53,54]. During DAH19-49, the juveniles fully established adult-like digestion. Here, protein-digesting enzymes, trypsin and pepsin, reached a peak, high AKP and LAP activity was observed, and AMS and LPS aligned with diet components; these reflect intestine maturation and the efficient application of formulated diets [55,56,57,58,59].

For the olive flounder (*Paralichthys olivaceus*), low fishmeal substitution with insect meal (black soldier fly) and/or other protein alternatives (FM35–45) did not result in disease after feeding [60] despite potential changes in gut microbes. Growth and health measurements of olive flounder in experimental groups were the same as those from the control group consuming 70% fishmeal, indicating a gut response to dietary reformulation [60]. These results, in contrast to *L. crocea* where dietary changes resulted in rapid enzymatic and metabolic shifts, especially in larval stages, highlight increased developmental sensitivity. For juvenile southern catfish (*Silurus meridionalis*), formulated diets resulted in higher peak metabolic rates, faster gastric drainage, and a greater specific growth rate compared to those fed natural prey (loach meat), and the discrepancies were linked to macronutrient equilibrium and microbial community diversity [61]. Likewise, *L. crocea* demonstrated developmental stage-dependent digestion optimized for natural prey, which was slowly modified under formulated diets. In contrast to *S. meridionalis*, *L. crocea* showed progressive dietary transitions, indicative of species-specific digestive plasticity. Larvae of European seabass (*Dicentrarchus labrax*) exhibited adaptive feeding by transitioning from live food to microparticulate diets in response to increased availability of microparticulate feed in their environment [62]. As a result, decreased growth rates initially led to enzyme (synthetic) upregulation, but this response eventually enabled adaptation. Similarly, an induction with enzymes facilitated the transition to formulated diets in *L. crocea*, although the mortality risk was high during this time. Enzyme activity exhibited these developmental stage-specific patterns including early endogenous control, mid-stage diet stimulation, and late convergence to digestion as an adult. The increased enzyme activities facilitated rapid growth rates and provided direction for the formulation of feed and larval survival.

### 4.2. Transcriptome Enrichment Pathway Analyses

In *L. crocea*, the metabolic pathways, vital for energy production and biosynthesis, were enriched throughout development. In the early developmental stages, larvae relied on yolk proteins/lipids, which comprised low enzyme activity [63,64,65]. At the onset of feeding bile secretion, cAMP signaling, DNA replication, and cell cycle pathways were enriched, highlighting proliferation through increased protein intake. During DAH3–19, live feed (rotifers, *Artemia*, and copepods) supplied high-quality proteins to the diet [66,67], resulting in an increase in AMS activity, increasing carbohydrate use. During this stage, enriched pathways included steroid biosynthesis, fat digestion, antigen presentation, and cell cycle regulation, representing immune and endocrine maturation. By DAH49, the larvae had matured digestive systems; AMS enabled carbohydrate use, thereby sparing proteins, lipids provided polyunsaturated fatty acids (PUFAs) for neural and immune functions [68], and enriched insulin secretion and olfactory transduction reflected the energy balance and adaptive feeding behavior [69]. Thus, the pathways shifted from yolk mobilization (early stage) to exogenous protein/lipid use with immune development (mid stage) to efficient carbohydrate utilization and energy regulation (late stage). These developmental stages matched enzyme activity changes with basal activity in early stages (DAH3–7), protease-dominated activity in the mid stage (DAH7–19), and carbohydrate/lipid digestion in the late stage (DAH19–49). Together, transcriptome analyses highlighted developmental stage-dependent nutritional adaptation. A cross-species comparison of fishes indicated that carnivores have increased growth rates, gastrointestinal evacuation, and postprandial metabolic responses compared to omnivores and herbivores [70]. Omnivores, by contrast, have a greater diversity of intestinal microbes. For *L. crocea*, the increased early upregulation of proteases is consistent with that in other carnivorous fish species, while subsequent increases in AMS and LPS indicated omnivorous digestion adaptation to ensure easy consumption of formulated meals.

### 4.3. Nutritional Adaptation from a Metabolomic Perspective

This study demonstrated that the metabolic profiles of larvae and juveniles of the NZ stock of large yellow croaker exhibited clear developmental stage-specific characteristics across feeding regimes, suggesting that metabolic regulation is driven by digestive system development. Generally, differential metabolites were mainly enriched in pathways related to amino acid, lipid, carbohydrate, nucleotide, and cofactor/vitamin metabolism, consistent with the high energy supply and biosynthetic demands of young Atlantic salmon (*Salmo salar*) and grouper (*Epinephelus* spp.) [71,72,73,74,75].

During the early developmental stage, larvae were dependent on yolk nutrients and had limited digestive function. The significantly enriched pathways included DNA replication, the cell cycle, and bile secretion, reflecting the mobilization of energy and substrates supporting rapid cell division and preparation for exogenous feeding. Active amino acid metabolism underscores the central role of proteins in cell proliferation and tissue formation and suggests that essential amino acids may function as energy substrates during this period. This finding is consistent with other species, such as Atlantic cod (*Gadus morhua*) and European seabass (*Dicentrarchus labrax*), where essential amino acids served as key energy sources during early development [76,77,78,79,80,81]. Combined with low digestive enzyme activity, it can be inferred that metabolism, at this stage, was primarily genetically regulated, ensuring mobilization and utilization of endogenous reserves.

At the onset of live feeding (DAH3–19), metabolites were significantly enriched in pathways related to fat digestion and absorption, steroid biosynthesis, and antigen processing and presentation, indicating that the metabolic profiles were shaped by exogenous protein and lipid intake. Increases in trypsin and pepsin were observed, consistent with the enrichment of protein digestion and absorption pathways. The activation of lipid-related pathways highlights the importance of unsaturated fatty acids from live feeds for membrane synthesis, hormone production, and immune development [82,83,84]. These results are consistent with those from other populations of large yellow croaker [18] where amino acid and lipid metabolism influenced rapid growth and immune system maturation during live feeding stages.

By the formulated diet feeding stage (DAH49), metabolomic profiles were significantly enriched in pathways including carbon metabolism, insulin secretion, and energy regulation, reflecting digestive system adaptation to the nutritional composition of formulated diets. Increased AMS and LPS activities indicated enhanced carbohydrate and lipid digestive capacity, with carbohydrates becoming increasingly important energy sources, thereby contributing to the protein-sparing effect. Similar findings have been reported in rainbow trout (*Oncorhynchus mykiss*), where enhanced carbohydrate metabolism during formulated diet feeding reduced protein use for energy, thus improving growth efficiency [85,86,87]. Furthermore, olfactory transduction pathway enrichment suggests that larvae may rely on sensory mechanisms to adjust feeding behavior, improving adaptation to formulated diets.

This study revealed developmental stage-dependent nutritional shifts in larvae and juveniles of the NZ stock of large yellow croaker. Early larval stages were dependent on endogenous proteins and lipids for energy. During the mid-developmental stages, they mainly utilized exogenous protein and phosphorus balanced by the inclusion of some lipids, with the gradual development of immunity. During the later developmental stages, they showed improved carbohydrate digestion and energy dispensation, adapting well to the formulated diet. These shifts also revealed coordinated changes between digestive enzyme activity and metabolic pathways, indicating that metabolomics is a valuable tool for evaluating nutritional needs of fish and for future diet adaptation. Importantly, these findings suggest that gene expression changes concurrently observed with dietary changes may directly alter nutrient utilization and energy supply. Furthermore, when diets are changed, the body responds within minutes at the mRNA level and by producing gene products like digestive enzymes. During the transition from endogenous to exogenous feeding, transcriptomics showed a cross-stage expression of DEGs with enriched protein and fat digestion. This approach also identified multiple DEGs over the different feeding periods and the transition from endogenous to exogenous feeding involved DEGs enriched in protein and fat digestion, the cell cycle, and energy metabolism pathways. Metabolomics results were consistent with these findings, with stage-specific changes in amino acid, lipid, and derivative levels observed, with overlapping KEGG pathways. This agreement between gene expression and metabolite levels demonstrated that dietary changes activated digestion- and metabolism-related genes and directly altered nutrient utilization and energy supply. For example, from DAH7 to DAH19, protein and lipid digestion genes were upregulated with shifts in amino acid and bile acid metabolites, and from DAH19 to DAH49, active carbon metabolism and energy regulation were indicated by transcriptomics and increased carbohydrate utilization, and energy-related metabolites were observed through metabolomics.

Collectively, these comparisons underscore the similarities and differences in digestive physiology between fish species. Like olive flounder, *L. crocea* exhibits enzymatic adaptation, but unlike yellow catfish (*Pelteobagrus fulvidraco*) or flounder, the larval stages are exceptionally sensitive to food adaptation. A study on seabass larvae indicated that a functional enzymatic system does not always translate to growth success unless the artificial diet is nutritional and palatable. Furthermore, genera are studied at different scales implying that digestive enzyme maturation, the degree of host microbiota modulation, and specific dynamic actions are determining factors for aquaculture performance. For *L. crocea*, adjusting the feed to suit the developmental digestive stage may decrease mortality rates, improve seed quality, and enhance aquaculture sustainability.

## 5. Conclusions

This study systematically investigated the digestive physiology and nutritional adaptation of larvae and juveniles of the NZ stock of large yellow croaker across five representative feeding stages. By integrating digestive enzyme assays, transcriptomic profiling, and metabolomic analyses, we revealed developmental stage-dependent changes in digestive capacity and metabolic regulation. Early larvae primarily relied on yolk reserves, supported by low but detectable levels of digestive enzymes and enrichment of pathways related to cell proliferation and yolk mobilization. At the onset of exogenous feeding, protease activities increased, and protein and lipid metabolism pathways were significantly enriched, highlighting the critical role of dietary proteins and lipids for rapid growth and immune development. In later stages, AMS and LPS activities were enhanced, and carbohydrate and energy regulation pathways were enriched, reflecting adaptation to formulated diets and the establishment of a mature digestive system.

The consistency between the transcriptomic and metabolomic results demonstrated that dietary transitions induced transcriptional regulation and metabolite-level changes, providing a comprehensive perspective of the molecular and physiological mechanisms of nutritional adaptation. These findings deepen our understanding of digestive and metabolic strategies of *L. crocea* during early development and offer practical guidance for hatchery practices. This study has provided a scientific basis for developmental stage-specific diet design, feeding regime optimization, methods for larval survival improvement, and efficient transition from live prey to formulated feeds. Collectively, this work contributes to advancing seedling production efficiency and supports the sustainable development of large yellow croaker aquaculture farms.

## Figures and Tables

**Table 1:** Primer sequences for the RT-qPCR analysis to validate the RNA-seq.

Gene	Forward Primer	Reverse Primer
*GATM*	ACCTCCGACACCTCTGATTCC	TGGCATGACGAATGTTCACCTT
*aldh1l1*	CTACTTCGCTGGCTGGTGTGA	TTCAGTGCTGTCAGTGGAGTCA
*fabp3*	AGAGCACCGTCAAGAACACAGA	CTCCGAGTGTGAGTGTCAGTGT
*CYP24A1*	TCCACAAGAGCCTGAACACCAA	GCAGTCGTCTCAACTCCTCCAA
*COL10A1*	TGGTGAGCGAGGTGAGGATG	AGGTCCAGGCTTGCCAGTAG
*Ampd1*	AGTGCCAGAGACCGATGACAA	CACCGCTGATGCCTCTTACG
*ldhb*	GTGGTGGACAGTGCCTACGA	ATGGACACGGCTCATGTTCTTG
*Mep1a*	CCACAATCACCACCACCATACC	AGTCTGCGTTGTCCTGCTCAT
*Mep1b*	TGAATCGCTGCTCACAGAATGG	GCCGAGTCTCCTTCCTTACCT
*FBXO32*	CTCAGCGGAGTGGCACAGAA	CACAGCGAGATGTAGAGCGTCT
*β-actin*	TCGTCGGTCGTCCCAGGCAT	ATGGCGTGGGGCAGAGCGT

**Table 2:** Changes in digestive enzyme activities with different feed intake.

Enzyme Activity	Groups				
	DAH3	DAH7	DAH12	DAH19	DAH49
AKP (U/gprot)	178.20 ± 50.88 ^b^	277.96 ± 35.63 ^b^	433.56 ± 140.41 ^b^	200.44 ± 36.35 ^b^	1538.15 ± 418.13 ^a^
LAP (U/gprot)	27.46 ± 2.57 ^b,c^	31.61 ± 2.00 ^b^	33.59 ± 4.78 ^b^	21.72 ± 1.90 ^c^	115.72 ± 6.37 ^a^
LPS (U/gprot)	9.64 ± 3.90 ^a,b^	4.74 ± 1.96 ^b^	8.08 ± 3.13 ^a,b^	6.94 ± 1.46 ^b^	12.61 ± 2.52 ^a,b^
AMS (U/mgprot)	0.57 ± 0.32 ^c^	0.92 ± 0.21 ^b,c^	2.19 ± 0.60 ^b^	0.53 ± 0.07 ^c^	7.04 ± 1.47 ^a^
pepsin (U/mgprot)	6.20 ± 1.22 ^b,c^	7.55 ± 1.14 ^b^	6.32 ± 1.04 ^b,c^	4.82 ± 0.85 ^c^	10.08 ± 0.33 ^a^
trypsin (U/mgprot)	3932.00 ± 980.17 ^b^	5974.55 ± 1007.40 ^b^	5663.87 ± 1203.41 ^b^	5602.83 ± 625.11 ^b^	20,868.33 ± 3721.35 ^a^

Note: data are expressed as mean ±  SD (*n*  = 3), and values with different superscript letters in the same row are statistically significant (*p* < 0.05).

**Table 3:** The transcriptome data of each sample.

Sample	RawDatas (**bp**)	CleanData (bp)	AF_Q20 (%)	AF_Q30 (%)	AF_GC (%)
DAH3-1	43,799,672	43,797,018	99.26%	97.37%	48.04%
DAH3-2	46,815,866	46,812,652	99.41%	97.76%	47.96%
DAH3-3	44,791,380	44,789,222	99.41%	97.75%	47.75%
DAH7-1	44,513,136	44,510,960	99.41%	97.75%	47.94%
DAH7-2	43,796,714	43,794,284	99.38%	97.66%	48.64%
DAH7-3	41,764,204	41,762,120	99.36%	97.57%	47.96%
DAH12-1	49,307,382	49,304,472	99.42%	97.76%	48.54%
DAH12-2	42,782,846	42,780,762	99.37%	97.62%	48.39%
DAH12-3	52,675,464	52,672,898	99.39%	97.68%	48.77%
DAH19-1	51,524,090	51,521,534	99.46%	97.95%	48.64%
DAH19-2	41,108,812	40,925,070	99.03%	97.03%	47.60%
DAH19-3	47,167,370	47,163,990	99.43%	97.82%	48.28%
DAH49-1	42,630,398	42,627,970	99.48%	98.00%	48.09%
DAH49-2	43,406,286	43,403,608	99.44%	97.87%	47.84%
DAH49-3	40,654,842	40,653,052	99.42%	97.78%	47.63%

**Table 4:** Key differential metabolites (DAH3–DAH7).

ID	MS2_Name	log2_FC	*p*-Value	VIP
M514T190_3_neg_NEG	Taurocholate	9.32	0.00	24.20
M498T150_2_neg_NEG	Taurochenodeoxycholate	8.17	0.00	18.41
M301T38_neg_NEG	8S-Hydroxy-5Z,9E,11Z,14Z-eicosatetraenoic acid	1.30	0.00	12.04
M367T300_2_neg_NEG	3-O-Feruloylquinic acid	6.65	0.00	11.81
M130T259_1_neg_NEG	Isoleucine	1.61	0.00	10.40
M255T38_2_neg_NEG	Palmitic acid	−0.93	0.00	9.63
M124T292_2_neg_NEG	Taurine	1.37	0.00	9.02
M164T253_3_neg_NEG	N-Acetyl-L-phenylalanine	1.49	0.00	8.89
M281T38_2_neg_NEG	Oleic acid	0.82	0.00	8.69
M496T189_1_pos_POS	1-Palmitoyl-sn-glycero-3-phosphocholine	1.56	0.00	10.80
M132T345_3_pos_POS	Creatine	0.24	0.00	8.55
M568T181_1_pos_POS	1-Stearoyl-2-hydroxy-sn-glycero-3-phosphocholine	−1.05	0.00	7.00

**Table 5:** Key differential metabolites (DAH7–DAH12).

ID	MS2_Name	log2_FC	*p*-Value	VIP
M137T167_3_pos_POS	Hypoxanthine	0.62	0.00	10.29
M104T271_2_pos_POS	Choline cation	−0.56	0.00	10.08
M496T189_1_pos_POS	1-Palmitoyl-sn-glycero-3-phosphocholine	−0.42	0.00	7.89
M136T159_1_pos_POS	Adenine	−0.72	0.00	7.84
M132T259_1_pos_POS	Leucine	−0.79	0.00	5.99
M482T195_pos_POS	1-Hexadecyl-sn-glycero-3-phosphocholine	−2.33	0.00	5.93
M76T329_3_pos_POS	Trimethylamine N-oxide	−0.28	0.00	5.79
M86T259_pos_POS	1,5-Pentanediamine	−0.83	0.00	5.70
M514T190_3_neg_NEG	Taurocholate	−1.654	0.00	23.40
M301T38_neg_NEG	8S-Hydroxy-5Z,9E,11Z,14Z-eicosatetraenoic acid	−1.89	0.00	15.63
M498T150_2_neg_NEG	Taurochenodeoxycholate	−0.90	0.00	14.70
M218T267_2_neg_NEG	Pantothenate	−0.98	0.00	4.52

**Table 6:** Key differential metabolites (DAH12–DAH19).

ID	MS2_Name	log2_FC	*p*-Value	VIP
M132T259_1_pos_POS	Leucine	−0.63	0.00	4.73
M123T61_pos_POS	Niacinamide	−1.19	0.00	4.68
M204T304_1_pos_POS	Acetyl-DL-carnitine	0.54	0.00	9.01
M159T167_pos_POS	Vitamin C	1.30	0.00	2.98
M377T35_pos_POS	Lithocholic acid	−2.59	0.00	1.69
M1030T190_neg_NEG	Taurocholic acid	1.86	0.00	2.78
M218T267_2_neg_NEG	Pantothenate	−0.38	0.00	2.76
M496T37_neg_NEG	Microcystin LR	3.56	0.00	2.89
M277T173_neg_NEG	Leu-Phe	−5.90	0.00	3.37
M159T320_neg_NEG	Ala-Ala	−5.56	0.00	3.77
M117T374_neg_NEG	Succinic acid	−1.44	0.00	3.73
M464T184_neg_NEG	Glycocholic acid	−1.84	0.00	3.61

**Table 7:** Key differential metabolites (DAH19–DAH49).

ID	MS2_Name	log2_FC	*p*-Value	VIP
M123T61_pos_POS	Niacinamide	2.97	0.00	5.43
M466T197_2_pos_POS	Glycocholate	7.38	0.00	5.00
M76T329_3_pos_POS	Trimethylamine N-oxide	0.63	0.00	4.81
M377T35_pos_POS	Lithocholic acid	5.46	0.00	2.68
M786T117_pos_POS	Deoxycholic acid	−1.45	0.00	2.57
M265T368_pos_POS	Thiamine cation	2.53	0.00	1.84
M345T255_pos_POS	11-Dehydrocorticosterone	5.20	0.00	1.52
M498T150_2_neg_NEG	Taurochenodeoxycholate	2.27	0.00	22.35
M514T190_3_neg_NEG	Taurocholate	2.42	0.00	21.48
M130T259_1_neg_NEG	Isoleucine	3.57	0.00	13.10
M434T35_neg_NEG	Paxilline	7.79	0.00	7.09
M124T292_2_neg_NEG	Taurine	1.07	0.00	7.00

## Data Availability

Upon a reasonable request, the corresponding author will provide the data supporting the results of this study.

## Supplementary Materials

The following supporting information can be downloaded at: https://www.mdpi.com/article/10.3390/ani16010120/s1, Table S1: Proximate composition of the experimental diet.

## References

[1] Zhang Y.Q., Guo H.Y., Liu B.S., Zhang N., Zhu K.C., Zhang D.C. (2024). Analysis of morphological differences in five large yellow croaker (*Larimichthys crocea*) populations. Isr. J. Aquac.—Bamidgeh.

[2] Tian M., Xu G., Yu R. (1962). Geographic variation and population of morphological characteristics of *Pseudosciaena crocea* (Richardson). Stud. Mar Sin..

[3] Xu G., Tian M., Zheng W. (1963). The stocks of *Pseudosciaena crocea* Richardson. Proceedings of the Fourth Plenary Seminar of the Western Pacific Fisheries Research Commission.

[4] Chen B., Bai Y., Wang J., Ke Q., Zhou Z., Zhou T., Pan Y., Wu R., Wu X., Zheng W. (2023). Population structure and genome-wide evolutionary signatures reveal putative climate-driven habitat change and local adaptation in the large yellow croaker. Mar. Life Sci. Technol..

[5] Chen X., Miao L., He Q., Ke Q., Pu F., Li N., Zhou T., Xu P. (2024). Chromosome-level genome assembly for three geographical stocks of large yellow croaker (*Larimichthys crocea*). Sci. Data.

[6] Ministry of Agriculture and Rural Affairs of the People’s Republic of China (2023). Fishery and Fishery Administration Bureau. China Fishery Statistical Yearbook 2023.

[7] Liu B., Guo H., Liu B., Zhang N., Zhu K., Yan K., Sun J., Zhang D. (2024). Early development and allometric growth patterns of *Larimichthys crocea* (Richardson, 1846). Aquaculture.

[8] Wang H., Jin J., Amenyogbe E., Liu Y., Huang S., Lu Y., Wu R., Wang Z., Boamah G.A., Huang J. (2025). Chromosome-level genome assembly and evolutionary analysis of the Nao-zhou stock of large yellow croaker (*Larimichthys crocea*). Genomics.

[9] Wang H.-J., Huang S.-P., Amenyogbe E., Liu Y., Jin J.-H., Lu Y., Boateng C.N., Wang Z.-L., Huang J.-S. (2025). Comprehensive Whole-Genome Survey and Analysis of the Naozhou Stock of Large Yellow Croakers (*Larimichthys crocea*). Animals.

[10] Cheng S., Du C., Zhu J.Q., Wu X.F. (2014). Embryonic and larval development of large yellow croaker (*Pseudosciaena crocea*) in Daiquyang. Adv. Mar. Sci..

[11] Chen R., Hong Y., Hong Y., Sun K., Hong Y. (2018). Study on formulated diet for large yellow croaker (*Larimichthys crocea*) larvae at the early feeding stage. Iran. J. Fish. Sci..

[12] Ma H., Cahu C., Zambonino J., Yu H., Duan Q., Le Gall M., Mai K. (2005). Activities of selected digestive enzymes during larval development of large yellow croaker (*Pseudosciaena crocea*). Aquaculture.

[13] Mai K., Yu H., Ma H., Duan Q., Gisbert E., Zambonino Infante J.L., Cahu C.L. (2005). A histological study on the development of the digestive system of *Pseudosciaena crocea* larvae and juveniles. J. Fish Biol..

[14] Xu X.J., Wang J., Xie Y.J., Su Y.Q. (2010). Histological study on postembryonic development of digestive system in large yellow croaker (*Pseudosciaena crocea*). J. Dalian Fish. Univ..

[15] Zhang Y.L., Zhang H.L., Wang L.Y., Gu B.Y., Fan Q.X. (2017). Research progress on allometric growth, nucleic acid, and digestive enzyme changes in fish during early developmental stages. J. Fish. Sci. China.

[16] Huang W., Yao C., Liu Y., Xu N., Yin Z., Xu W., Miao Y., Mai K., Ai Q. (2020). Dietary Allicin Improved the Survival and Growth of Large Yellow Croaker (*Larimichthys crocea*) Larvae via Promoting Intestinal Development, Alleviating Inflammation and Enhancing Appetite. Front. Physiol..

[17] Pan Y., Dahms H., Hwang J., Souissi S. (2022). Recent Trends in Live Feeds for Marine Larviculture: A Mini Review. Front. Mar. Sci..

[18] Liu J.F. (1999). Study on embryonic development of large yellow croaker (*Pseudosciaena crocea*) and morphological characteristics and ecological habits of its larvae and juveniles under artificial seedling conditions. Mar. Sci..

[19] Yu H.R., Mai K.S., Duan Q.Y., Ma H.M., Liufu Z.G., Tan B.P. (2003). Feeding habits and growth performance of larvae and juveniles of *Pseudosciaena crocea* under artificial rearing conditions. J. Fish. Sci. China.

[20] Zheng L., Zeng C., Zhu W., Zhang J., Wang L., Shao J., Zhao W. (2024). TLR2/TLR5 Signaling and Gut Microbiota Mediate Soybean-Meal-Induced Enteritis and Declined Growth and Antioxidant Capabilities in Large Yellow Croaker (*Larimichthys crocea*). J. Mar. Sci. Eng..

[21] Zhu W., Yuan X., Luo H., Shao J., Chen X. (2021). High percentage of dietary soybean meal inhibited growth, impaired intestine healthy and induced inflammation by TLR-MAPK/NF-κB signaling pathway in large yellow croaker (*Larimichthys crocea*). Aquac. Rep..

[22] Pang A., Peng C., Xie R., Wang Z., Tan B., Wang T., Zhang W. (2023). Effects of fermented soybean meal substitution for fish meal on intestinal flora and intestinal health in pearl gentian grouper. Front. Physiol..

[23] Wang P., Zhou Q., Feng J., He J., Lou Y., Zhu J. (2019). Effect of dietary fermented soybean meal on growth, intestinal morphology and microbiota in juvenile large yellow croaker, *Larimichthys crocea*. Aquac. Res..

[24] Fu R., Wang G., Li X., Zhu X., Ren P., Zhang L., Ai Q., Wang Z. (2025). Decoding feed efficiency: Liver and intestinal transcriptomics in *Larimichthys crocea* fed a fishmeal-free diet. Aquac. Fish..

[25] Ke Q., Li Y., Weng H., Chen B., Wang J., Zhao J., Jiang P., Xu P., Zhou T. (2025). Differential responses of the intestine and liver transcriptome to high levels of plant proteins in diets for large yellow croaker (*Larimichthys crocea*). Front. Genet..

[26] Zhao J., Ai Q., Mai K., Zuo R., Luo Y. (2013). Effects of dietary phospholipids on survival, growth, digestive enzymes and stress resistance of large yellow croaker, *Larmichthys crocea* larvae. Aquaculture.

[27] Xu W., Wang Y., Zhang J., Liu J., Liu Y., Huang W., Yao C., Mai K., Ai Q. (2024). Dietary Oryzanol (Ory) Improved the Survival and Growth of Large Yellow Croaker (*Larimichthys crocea*) Larvae via Promoting Activities of Digestive Enzymes, Antioxidant Capacity, and Lipid Metabolism. Aquac. Nutr..

[28] Yu H.R. (2006). Study on Digestive Physiology and Requirements for Protein and Methionine in Larvae and Juveniles of Large Yellow Croaker (*Pseudosciaena crocea*). Ph.D. Thesis.

[29] China Sustainable Seafood Assessment (CSSA) (2024). Large Yellow Croaker Assessment Brief.

[30] (2019). FAO Chinese Production and Export of Large Yellow Croaker. GLOBEFISH News. https://www.fao.org/in-action/globefish/news-events/news/news-detail/chinese-production-and-export-of-large-yellow-croaker-/en.

[31] Haendiges J., Timme R., Ramachandran P., Balkey M. (2020). DNA Quantification Using the Qubit Fluorometer.

[32] Love M.I., Huber W., Anders S. (2014). Moderated estimation of fold change and dispersion for RNA-seq data with DESeq2. Genome Biol..

[33] Livak K.J., Schmittgen T.D. (2001). Analysis of relative gene expression data using real-time quantitative PCR and the 2−ΔΔCT method. Methods.

[34] Yang E., Amenyogbe E., Zhang J., Wang W., Huang J., Chen G. (2022). Integrated transcriptomics and metabolomics analysis of the intestine of cobia (*Rachycentron canadum*) under hypoxia stress. Aquac. Rep..

[35] Smith C.A., Want E.J., O’Maille G., Abagyan R., Siuzdak G. (2006). XCMS: Processing mass spectrometry data for metabolite profiling using nonlinear peak alignment, matching, and identification. Anal. Chem..

[36] Do K.T., Wahl S., Raffler J., Molnos S., Laimighofer M., Adamski J., Suhre K., Strauch K., Peters A., Gieger C. (2018). Characterization of missing values in untargeted MS-based metabolomics data and evaluation of missing data handling strategies. Metabolomics Off. J. Metabolomic Soc..

[37] Sun J., Xia Y. (2024). Pretreating and normalizing metabolomics data for statistical analysis. Genes Dis..

[38] Hao R., Du X., Yang C., Deng Y., Zheng Z., Wang Q. (2019). Integrated application of transcriptomics and metabolomics provides insights into unsynchronized growth in pearl oyster *Pinctada fucata* martensii. Sci. Total Environ..

[39] Liu F., Li S., Yu Y., Sun M., Xiang J., Li F. (2020). Effects of ammonia stress on the hemocytes of the Pacific white shrimp *Litopenaeus vannamei*. Chemosphere.

[40] Kolkovski S., Tandler A., Izquierdo M.S. (1997). Effects of live food and dietary digestive enzymes on the efficiency of microdiets for seabass (*Dicentrarchus labrax*) larvae. Aquaculture.

[41] Munilla-Moran R., Stark J.R., Barbour A. (1990). The role of exogenous enzymes in digestion in cultured turbot larvae (*Scophthalmus maximus* L.). Aquaculture.

[42] García-Ortega A., Verreth J., Coutteau P., Segner H., Huisman E., Sorgeloos P. (1998). Biochemical and enzymatic characterization of decapsulated cysts and nauplii of the brine shrimp Artemia at different developmental stages. Aquaculture.

[43] Lovett D.L. (1989). Ontogeny of gut morphology in the white shrimp *Penaeus setiferus* (Decapoda, Penaeidae). J. Morphol..

[44] Lazo C.R., Holt G.J., Arnold C.R. (2015). Ontogeny of pancreatic enzymes in larval red drum *Sciaenops ocellatus*. Aquac. Nutr..

[45] Sun M., Chai X.J., Xu Y.J., Wang Y.B., Hu Z.H. (2012). Study on changes of digestive enzyme activities during early development of yellow drum (*Nibea albiflora*). J. Shanghai Ocean Univ..

[46] Xu A., Liu X.Y., Zhang Y., Pan P., Sun D.J. (2014). Distribution and activity of digestive enzymes in female broodstock of *Acipenser ruthenus* at different gonadal development stages. J. Dalian Ocean Univ..

[47] Chen B.N., Qin J.G., Kumar M.S., Hutchinson W.G., Clarke S.M. (2006). Ontogenetic development of digestive enzymes in yellowtail kingfish (*Seriola lalandi*) larvae. Aquaculture.

[48] Gisbert E., Giménez G., Fernández I., Kotzamanis Y., Estévez A. (2009). Development of digestive enzymes in common dentex *Dentex dentex* during early ontogeny. Aquaculture.

[49] Su Z.X., Yue Y.F., Shi Z.H., Peng S.M., Xia L.J. (2021). Changes in digestive enzyme and non-specific immune factor activities during larval and juvenile development of *Diodon holocanthus*. Mar. Fish..

[50] Liu M., Gao Y. (2012). Study on changes of protease activity during larval and juvenile development of yellow catfish (*Pelteobagrus fulvidraco*). Hebei Fish..

[51] Pan L., Fang H., Zhang S.C., Wang X., Jian Y.X., Hu F.W., Gao F.X., Guo W. (2013). Changes of digestive enzyme activities in larval, juvenile, and young *Hexagrammos otakii*. Prog. Fish. Sci..

[52] Yan T.M., Li S., He Z.D., He L., Ruan H.B., He Z. (2019). Intestinal morphological development and digestive enzyme activities of larval and juvenile *Percocypris pingi*. J. Sichuan Agric. Univ..

[53] Huang X.B., Li G.F. (2007). Research status of nutritional requirements for larval and juvenile fish. Feed. Res..

[54] Lin J.B. (2017). Research progress on nutrition and feed of marine fish larvae and juveniles (I). Feed. Husb..

[55] Conklin D.E., Piedrahita R.H., Merino G.E., Muguet J.-B., Bush D.E., Gisbert E., Rounds J., Cervantes-Trujano M. (2004). Development of California halibut, *Paralichthys californicus*, culture. J. Appl. Aquac..

[56] Infante J.Z., Cahu C. (2001). Ontogeny of the gastrointestinal tract of marine fish larvae. Comp. Biochem. Physiol. Part C Toxicol. Pharmacol..

[57] 57, Kvåle A., Mangor-Jensen A., Moren M., Espe M., Hamre K. (2007). Development and characterisation of some intestinal enzymes in Atlantic cod (*Gadus morhua* L.) and Atlantic halibut (*Hippoglossus hippoglossus* L.) larvae. Aquaculture.

[58] Ribeiro L., Zambonino-Infante J., Cahu C., Dinis M. (1999). Development of digestive enzymes in larvae of *Solea senegalensis*, Kaup 1858. Aquaculture.

[59] Suzer C., Aktülün S., Çoban D., Okan K.H., Saka Ş., Fırat K., Alpbaz A. (2007). Digestive enzyme activities in larvae of sharpsnout seabream (*Diplodus puntazzo*). Comp. Biochem. Physiol. Part A Mol. Integr. Physiol..

[60] Jo S., Park S., Lee S., Tran B.T., Kim J.S., Song J., Lee B., Hur S., Nam T., Lee K. (2021). Effect of low-fishmeal diets on some digestive physiological responses of juvenile and growing olive flounder (*Paralichthys olivaceus*) fed at an industrial-scale fish farm. Aquac. Rep..

[61] Pang X., Tan G., Sun H., Shi H.Y., Su S.Q., Li Y., Fu S.J. (2022). Effect of feeding different diets on postprandial metabolic response, digestive capacity and growth performance in juvenile southern catfish (*Silurus meridionalis*). Aquac. Rep..

[62] Infante J.L.Z., Cahu C. (1994). Development and response to a diet change of some digestive enzymes in sea bass (*Dicentrarchus labrax*) larvae. Fish Physiol. Biochem..

[63] Anderson J.L., Carten J.D., Farber S.A. (2011). Zebrafish lipid metabolism: From mediating early patterning to the metabolism of dietary fat and cholesterol. Methods Cell Biol..

[64] Fraher D., Sanigorski A., Mellett N.A., Meikle P.J., Sinclair A.J., Gibert Y. (2016). Zebrafish Embryonic Lipidomic Analysis Reveals that the Yolk Cell Is Metabolically Active in Processing Lipid. Cell Rep..

[65] Thiruvasagam T., Chidambaram P., Ranjan A., Komuhi N. (2024). Significance of fatty acids in fish broodstock nutrition. Anim. Reprod. Sci..

[66] Bougali S.B., Karakatsouli N., Ntomalis K., Kastelis A., Alexopoulou V., Batzina A., Markakis I. (2025). Effects of Co-Feeding Dry and Live Feed from the Onset of Exogenous Feeding on Red *Seabream Pagrus* major Larviculture and Pre-Growing. Fishes.

[67] Samat N.A., Yusoff F.M., Rasdi N.W., Karim M. (2020). Enhancement of Live Food Nutritional Status with Essential Nutrients for Improving Aquatic Animal Health: A Review. Animals.

[68] Al-Khalaifah H. (2020). Modulatory Effect of Dietary Polyunsaturated Fatty Acids on Immunity, Represented by Phagocytic Activity. Front. Vet. Sci..

[69] Matiashova L., Hoogkamer A.L., Timper K. (2023). The Role of the Olfactory System in Obesity and Metabolism in Humans: A Systematic Review and Meta-Analysis. Metabolites.

[70] Pang X., Yang J., Xiang S.A., Sun H., Fu S.J. (2024). Interspecific differences in growth, digestion, and feeding metabolism among freshwater fishes with different food habits. Front. Mar. Sci..

[71] Aguilar A., Mattos H., Carnicero B., Sanhueza N., Muñoz D., Teles M., Tort L., Boltaña S. (2022). Metabolomic Profiling Reveals Changes in Amino Acid and Energy Metabolism Pathways in Liver, Intestine and Brain of Zebrafish Exposed to Different Thermal Conditions. Front. Mar. Sci..

[72] Hao M., Zhu J., Xie Y., Cheng W., Yi L., Zhao S. (2024). Targeted metabolomics of muscle amino acid profles and hepatic transcriptomics analyses in grass carp (*Ctenopharyngodon idellus*) fed with broad beans. Heliyon.

[73] Ivanova L., Rangel-Huerta O.D., Tartor H., Dahle M.K., Uhlig S., Fæste C.K. (2024). Metabolomics and Multi-Omics Determination of Potential Plasma Biomarkers in PRV-1-Infected Atlantic Salmon. Metabolites.

[74] Liu Y., Li B., Hou Y., Zhou L., Yang Q., Zhang C., Li H., Zhu J., Jia R. (2025). Integrated Metabolomics and Transcriptomics Reveals Metabolic Pathway Changes in Common Carp Muscle Under Oxidative Stress. Antioxidants.

[75] Rajab S.A.S., Andersen L.K., Kenter L.W., Berlinsky D.L., Borski R.J., McGinty A.S., Ashwell C.M., Ferket P.R., Daniels H.V., Reading B.J. (2024). Combinatorial metabolomic and transcriptomic analysis of muscle growth in hybrid striped bass (female white bass *Morone chrysops* x male striped bass *M. Saxatilis*). BMC Genom..

[76] Goessling W., Sadler K.C. (2015). Zebrafish: An important tool for liver disease research. Gastroenterology.

[77] Herrera M., Herves M.A., Giráldez I., Skar K., Mogren H., Mortensen A., Puvanendran V. (2017). Effects of amino acid supplementations on metabolic and physiological parameters in Atlantic cod (*Gadus morhua*) under stress. Fish Physiol. Biochem..

[78] Khoklang A., Kersanté P., Nontasan S., Sutthi N., Pakdeenarong N., Wang T., Wangkahart E. (2024). Insights into the functional properties of a natural free amino acid mix: Effect on growth performance, nutrient metabolism, and immune response in a carnivorous fish, Asian seabass (*Lates calcarifer*). Fish Shellfish Immunol..

[79] Li P., Mai K., Trushenski J., Wu G. (2009). New developments in fish amino acid nutrition: Towards functional and environmentally oriented aquafeeds. Amino Acids.

[80] Salamanca N., Herrera M. (2024). Amino Acids as Dietary Additives for Enhancing Fish Welfare in Aquaculture. Animals.

[81] The Research Council of Norway (2009). The Fish Larva: A Transitional Life form, the Foundation for Aquaculture and Fisheries.

[82] Frías-Gómez S.A., Hernández Hernández L.H., Powell M.S., Álvarez-González C.A., Cortés-Jacinto E., Cigarroa-Ruiz L., Arellano-Carrasco G. (2023). Effect of dietary protein, lipid and carbohydrate ratio on growth, digestive and antioxidant enzyme activity of prawn *Macrobrachium acanthurus* postlarvae. Aquac. Rep..

[83] Torres M., Parets S., Fernández-Díaz J., Beteta-Göbel R., Rodríguez-Lorca R., Román R., Lladó V., Rosselló C.A., Fernández-García P., Escribá P.V. (2021). Lipids in Pathophysiology and Development of the Membrane Lipid Therapy: New Bioactive Lipids. Membranes.

[84] Wu J., Yu T., Wang Q., Zhang C., Fu D., Liu W., Jiang M., Xu L., Zhou Y., Wu J. (2024). Effects of dietary microbial protease on growth performance, nutrient apparent digestibility, hepatic antioxidant capacity, protease activities and intestinal microflora in juvenile genetically improved farmed tilapia, *Oreochromis niloticus*. Aquac. Rep..

[85] Brauge C., Medale F., Corraze G. (1994). Effect of dietary carbohydrate levels on growth, body composition and glycaemia in rainbow trout, *Oncorhynchus mykiss*, reared in seawater. Aquaculture.

[86] Groot R., Lyons P., Schrama J.W. (2021). Digestible energy versus net energy approaches in feed evaluation for rainbow trout (*Oncorhynchus mykiss*). Anim. Feed Sci. Technol..

[87] Hung S.S., Storebakken T. (1994). Carbohydrate Utilization by Rainbow Trout Is Affected by Feeding Strategy. J. Nutr..

