# Supplementary material for: Ontogenetic Changes in the Digestive Capacities of the Naozhou Stock of Large Yellow Croaker (Larimichthys crocea)
Source: Animals (Basel). 2025 Dec 31;16(1):120. doi: 10.3390/ani16010120 (PMC12785027; doi:10.3390/ani16010120)
Supplement: Supplementary file 1 [file animals-16-00120-s001.zip › animals-3991915-supplementary.pdf]

Table S1. Proximate composition of the experimental diet (%, dry matter basis)

**Item**	**Content (%)**
Fish meal	38.0
Soybean protein concentrate	14.0
Wheat flour	10.0
Corn gluten meal	6.0
Fish oil	7.0
Soybean oil	3.0
Dicalcium phosphate	1.2
Vitamin premix	1.2
Mineral premix	1.0
Choline chloride	0.4
Antioxidant	0.05
Other additives (e.g., β-glucan)	0.15
**Total**	**100.0**
Crude protein	52.7
Crude lipid	13.6
Crude fiber	3.4
Ash	11.5
Gross energy (kJ g^{-1})	21.0
